# Comparability across infrared spectroscopy techniques and substrates for bioanalytical applications

**DOI:** 10.1039/d6ra03641d

**Published:** 2026-08-27

**Authors:** Shuxia Guo, Supatcharee Cael, Samir F. El-Mashtoly, Christoph Krafft, Jürgen Popp, Thomas Bocklitz

**Affiliations:** a Institute of Physical Chemistry (IPC) and Abbe Center of Photonics (ACP), Friedrich Schiller University Jena, Member of the Leibniz Centre for Photonics in Infection Research (LPI) Helmholtzweg 4 07743 Jena Germany shuxia.guo@leibniz-ipht.de; b Leibniz Institute of Photonic Technology, Member of Leibniz Health Technologies, Member of the Leibniz Centre for Photonics in Infection Research (LPI) Albert-Einstein-Straße 9 07745 Jena Germany

## Abstract

The development of quantum cascade lasers (QCLs) brought new possibilities in infrared (IR) spectroscopy for bioanalytical applications, offering highly enhanced resolution, sensitivity, speed, and multi-modal capability. This has been supported by QCL-based IR techniques, including the laser direct infrared (LDIR) and optical photothermal IR (O-PTIR) methods. Despite their vast strengths, the comparability of these IR techniques with the established Fourier transform infrared (FTIR) spectroscopy technique remains an open question. In this study, we systematically evaluate and compare O-PTIR, LDIR, and FTIR spectroscopy using six representative biologically relevant samples (ATP, brain lipid extract, cholesterol, glycogen, lysozyme, and urea) and three commonly used substrates (CaF_2_, silicon, and MirrIR). The measurements were generally performed in the single-point mode, with brain lipid and urea measured additionally in the mapping mode to investigate the influence of sample heterogeneity. On this basis, we examined three sources of variability in this study: sample heterogeneity, IR technique, and substrate. The variability was characterized from the perspective of the band intensities, band positions, band widths, and spectral similarities based on the Pearson's correlation coefficient. Overall, the measurements demonstrate clear differences across both techniques and substrates. The LDIR measurements are largely different from FTIR and O-PTIR measurements, while CaF_2_ exhibited better stability than MirrIR (Kevley) and silicon. More importantly, we found that the substrate plays a role in band positions, while the variability across IR techniques does not show clear patterns. With this study, we expect to draw attention to the unavoidable spectral variability in IR measurements across both IR techniques and substrates. This should be carefully considered, especially in multi-center studies.

## Abstract 2

The comparability of IR measurements is investigated across IR techniques and substrates based on six representative samples with the support of statistical analysis.

## Graphical abstract

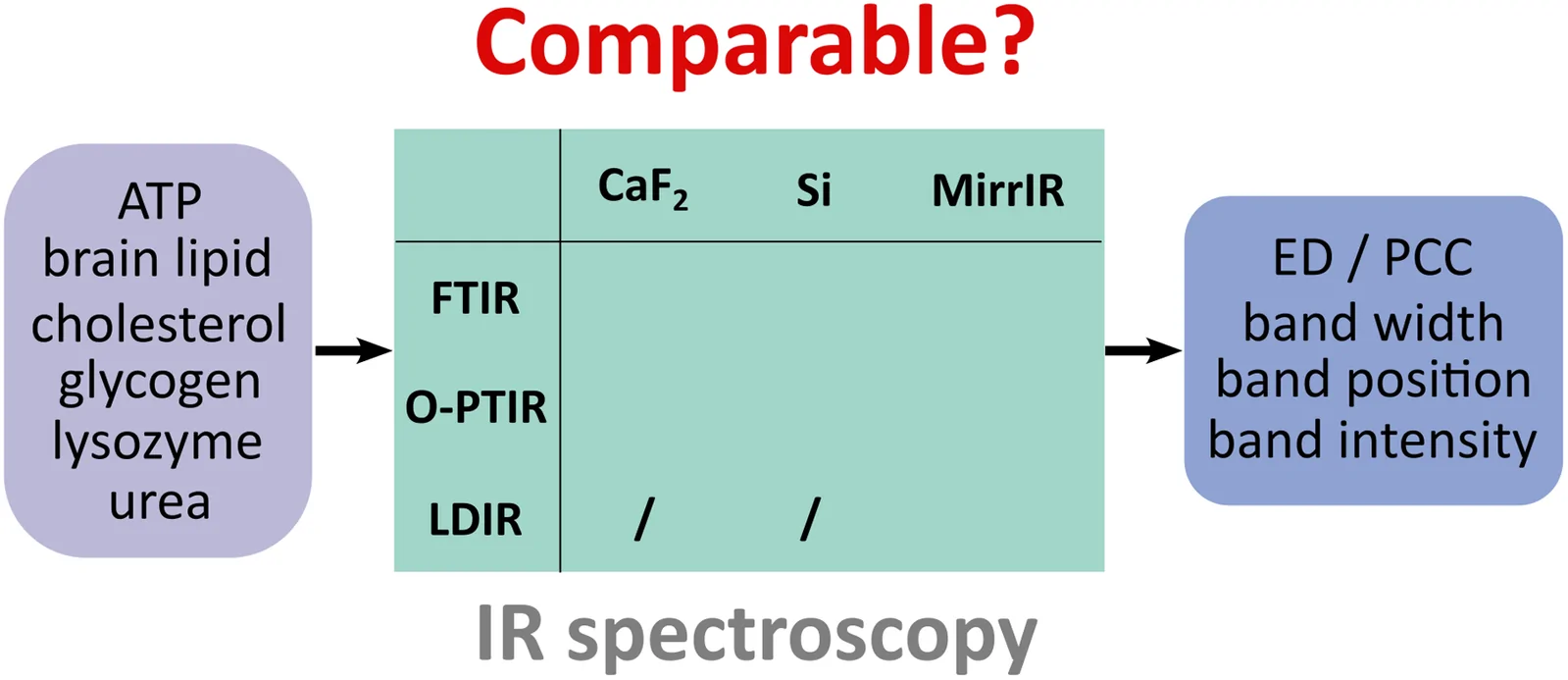

## 1. Introduction

Mid-infrared (IR) spectroscopy in the wavenumber range from 400 to 4000 cm^−1^ has found its place in a wide range of fields, including biomedical diagnostics,^1–3^ forensics,^4,5^ pharmaceuticals,^6,7^ and environmental science.^8,9^ By measuring the absorption of IR radiation by a sample, in which the wavenumber maximum represents the energy of IR-active vibrations, IR spectroscopy brings rich molecular information that can be considered as the fingerprint of the sample.^10^ This has led to IR spectroscopy becoming a popular analytical tool for non-invasive and label-free detection.^11,12^

Nowadays, IR spectra are mainly recorded using Fourier transform infrared (FTIR) spectroscopy.^12^ The principle is that broadband IR radiation is emitted by a thermal source (Globar), which is modulated by an interferometer. The interferogram passes the sample and is registered by an IR detector. Fourier transformation of the recorded signal generates the full spectrum. Due to its broad spectral coverage, accuracy and speed, FTIR has long been the dominant technique for IR spectroscopy. Its sensitivity and spatial resolution, however, remain limited because of the intrinsic nature of the incoherent light source in the mid-IR range.^13^

A technical breakthrough in IR spectroscopy was triggered by the development of quantum cascade lasers (QCLs),^14^ which provide tunable and high-power narrowband radiation within the mid-IR range (4–12 µm). This enables IR spectroscopy to be performed with compact, tunable laser sources, greatly enhancing the speed, resolution, and sensitivity of IR measurements. Several QCL-based IR instruments have been developed, including laser direct infrared spectroscopy (LDIR, Agilent),^9,15,16^ the Spero microscope (Daylight),^17^ the LUMOS II ILIM microscope (Bruker), and optical photothermal infrared spectroscopy (O-PTIR).^13,18–23^ The LDIR instruments are built upon precisely tunable QCLs, which enable IR spectroscopy to be operated under monochromatic infrared excitation with single-channel thermoelectrically cooled MCT detectors. The Spero and Lumos microscopes use microbolometer arrays for rapid discrete wavenumber imaging or hyperspectral imaging. This allows both discrete-frequency and full-range IR measurements when combined with rapid wavelength scanning. Thanks to the nature of QCLs as a coherent and high-power excitation source, LDIR is advantageous in terms of speed and sensitivity for discrete wavenumber imaging. Functioning within the mid-IR range, however, the spatial resolution of LDIR is fundamentally constrained by mid-IR diffraction, the low numerical apertures of IR-transparent lenses and windows, and the limits of thermal detectors.^24^ These constraints are largely bypassed in O-PTIR spectroscopy, another QCL-based IR technique. It utilizes two different lasers to detect an IR spectrum. The first laser, a pulsed pump mid-IR QCL, induces the photothermal effect upon IR absorption, leading to thermal expansion and changes in the refractive index in the sample. These photothermal effects change the light scattering of a probe laser, which is then detected *via* a photodetector. The application of the visible probe laser successfully bypasses the limitations of the IR optics, improving the spatial resolution to the sub-micron range. Furthermore, as the probe laser can simultaneously act as an excitation laser for Raman spectroscopy and fluorescence imaging, O-PTIR naturally enables Raman-IR-fluorescence multimodal measurements.

Besides the instruments, IR spectroscopy can be further complicated when taking into consideration the measurement mode, including transmission, diffuse reflection, specular reflection, and attenuated total reflectance (ATR). Each mode has advantages and disadvantages in depth of penetration, spectral quality, and sample compatibility; hence, it is best suited to certain sample types and analysis needs. The transmission mode is ideal for thin films or transparent materials; the transflection mode is often used for semi-transparent samples, like biological tissues; reflection mode is useful for opaque or thick samples; and the ATR mode is especially suitable for liquids, gels, and solids with rough surfaces. Besides the sample nature, it is important to note that the selection of measurement modes can be dependent on the IR instrument; for instance, O-PTIR and LDIR are typically operated in the reflection mode at the current stage.

As another factor, the substrate used in IR spectroscopy greatly affects the quality of a measured spectrum.^25,26^ The selection of a suitable IR substrate is dependent on both the sample and the measurement mode. Beyond the minimal IR absorption, refractive index (RI) is one of the most important criteria. For transmission IR, substrates with medium RI, such as CaF_2_, are preferred for a close match between the RI of the sample and substrate, which helps reduce spectral distortions from the sample-substrate interface, including interference and reflection. For reflection IR, in contrast, a very high RI substrate, like a metal, is used to ensure strong reflectivity. In the case of ATR, particularly, the refractive index of the prism must be higher than the refractive index of the sample.

With these considerations, the comparability of different IR measurements becomes critical for the application of IR spectroscopy. Several efforts have been made in this regard. For example, the O-PTIR measurements are shown to be highly correlated with the FTIR spectra in transmission mode,^21^ whereas IR spectroscopy in reflection, transflection, and ATR modes normally bears spectral distortions like band shifts, which are significantly more severe than in the transmission mode.^25,27–29^ For this reason, dedicated spectral libraries are required for transmission IR/O-PTIR spectra, ATR-IR spectra, and LDIR spectra. However, a systematic analysis to understand the variability across different IR techniques and measurement conditions is still lacking. We aim to fill this gap in this study. Six representative biologically relevant samples were measured with O-PTIR, LDIR, and FTIR spectroscopy, based on three commonly used substrates. Spectral variations were explored with respect to band positions, intensities, widths, and spectral similarities based on Pearson's correlation coefficients. A summary of the abbreviations used in this manuscript is given in Table S1. With this study, we aim to illustrate the spectral variabilities that are intrinsically contained across different IR techniques and substrates. We draw attention to such variability for possible approaches to standardization and to caution researchers who are undertaking multi-center and/or comparative studies.

## 2. Material and methods

### 2.1 Substances and sample preparation

This study analyzed six substances:^30–35^ ATP (Sigma, purity grade ≥99%), brain lipid extract (from bovine brain, Fluka, BioChemika grade), cholesterol (lanolin, Fluka, Purity grade ≥99%), glycogen (from bovine liver, Fluka, BioChemika grade), lysozyme (from hen egg white, BioChemika, 97 940 U mg^−1^), and urea (Carl Roth, purity grade ≥99.5%). Each substance was dissolved in either water or chloroform to reach a concentration of approximately 10 mg mL^−1^. A 5 µL drop of each solution was placed on CaF_2_, silicon (Si), and the silver-coated MirrIR slides (Kevley). Notably, before use, the Si surface was rinsed with ethanol to remove silicon dioxide (SiO_2_) and expose the underlying silanol (Si–OH) groups. The samples were then air-dried on the substrate completely before measurement. The diameter, thickness, and morphology of the dried films depend on the solvent used. Chloroform, used for lipids and cholesterol, did not form a droplet on the substrates; it evaporated rapidly and produced thinner layers. Water, used for the remaining substances, formed a droplet that took several minutes to dry. This resulted in thicker, but inhomogeneous, layers with a “coffee ring” at the margin. To accommodate this effect, we measured 3–5 spectra from each droplet at different positions and used the averaged spectra for analysis, as described in Section 2.2. The six substances were selected as representative model compounds covering major biochemical classes. This helps provide a general and controlled benchmark for evaluating the performance of the IR measurements based on chemically diverse systems with different functional groups, vibrational features, spectral complexity, signal strength, and background interference.

### 2.2 IR spectroscopy

**Fig. 1:**
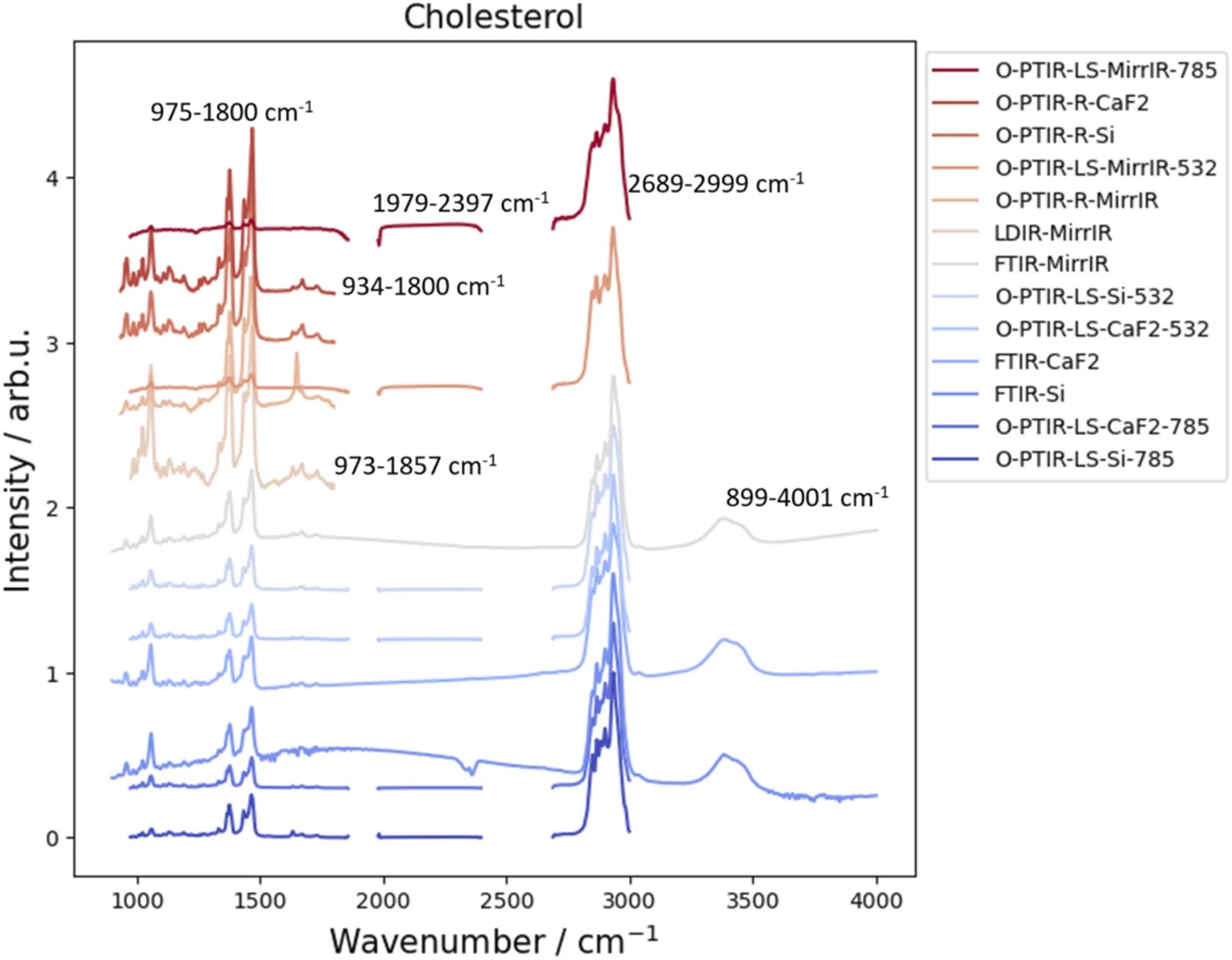
Overview of the IR spectra of cholesterol illustrating the wavenumber ranges of different measurements. Detailed plots of each substance are given in Fig. S1.

IR spectra were collected using three IR techniques: FTIR, O-PTIR, and LDIR. O-PTIR spectra at an indicated spectral resolution of 6.6 cm^−1^ were collected by Mirage microscopic instruments from Photothermal Spectroscopy Corp in reflection mode using all three substrates with 40×/NA 0.8 Cassegrain objective lenses, a scan number of 8, and a scan mode speed of 1000 cm^−1^ per s. Particularly, we performed the measurement in two settings featuring different probe wavelengths. For O-PTIR-LS, spectra were collected using a pump QCL laser covering the range 2999–973 cm^−1^ and probe lasers at 532 nm and 785 nm, while O-PTIR-R used a pump QCL laser covering the range of 2922–934 cm^−1^ and a 785 nm probe laser. This enabled us to evaluate the influence of the probe wavelength on signal response under otherwise identical O-PTIR conditions. FTIR spectra were obtained using a FTIR 670 spectrometer coupled to a FTIR 620 microscope (Agilent Technologies). Spectra were collected at a spectral resolution of 4 cm^−1^ with a 15×/NA 0.4 objective lens over the range of 4000–900 cm^−1^ using 16 background scans and 8 sample scans. Measurements were performed in transmission mode for CaF_2_ and Si substrates, and in reflection mode for MirrIR slides. LDIR spectra were acquired with an 8700 LDIR Chemical Imaging System (Agilent Technologies, California, USA) in reflection mode on MirrIR over the range of 1800–975 cm^−1^ with a resolution of 4 cm^−1^. We collected 3–5 spectra for all measurements, from which the average was used for the analysis. The typical IR bands of these substances are summarized accordingly in Table S2. The IR spectrum of cholesterol is shown in Fig. 1 to illustrate the spectral ranges of each measurement. Notably, we cannot fully synchronize the configurations of different IR techniques due to technical limitations. Among these, a spectral resolution of 6.6 cm^−1^ was used for O-PTIR, as it is not adjustable from the setup. A 4 cm^−1^ resolution was adopted for the other two techniques to ensure the best possible performance. The influence of the differences on the spectral resolutions will be discussed in the next section.

IR spectra were acquired in single-point mode for all six substances. Furthermore, to assess the effect of sample variation, the mapping mode was performed on brain lipid extract and urea samples. Without losing generalizability, brain lipid extract and urea were selected as representative examples solely to evaluate sample heterogeneity. The selection was not based on expected spectral behavior, and no systematic preference was intended. The mapping area of O-PTIR was used as a reference for other techniques. 12 × 10 hyperspectral O-PTIR maps (LS and R) were acquired over a 1100 × 900 µm^2^ area with a step size of 100 × 100 µm^2^ using the autofocus option before each spectral scan. A 3 × 3 mosaic of hyperspectral FTIR datasets comprising 192 × 192 spectra was collected over a 1050 × 1050 µm^2^ field of view. The spatial grid was subsequently resampled to a 10 × 10 pixel array in *X* and *Y* dimensions after import into the CytoSpec software,^36^ resulting in an interpolated spatial sampling interval of 105 × 105 µm^2^ per pixel. Hyperspectral LDIR images of 12 × 10 spectra were collected over a 1200 × 1000 µm^2^ area with a step size of 100 × 100 µm^2^.

To make the description clearer, we refer to a measurement as IR spectroscopy with a certain instrument and substrate in the rest of this manuscript.

### 2.3 Data analysis

#### 2.3.1 Spectral preprocessing

To compare the spectral range of different IR techniques, all spectra were cut into 975–1800 cm^−1^ as the first step of the analysis. All spectra were interpolated onto an interval of 1 cm^−1^. Thereafter, the statistics-sensitive non-linear iterative peak-clipping (SNIP) algorithm was employed with parameters iterations = 40, order = 2 to reduce the baseline profiles underneath the IR bands, which result from non-absorptive signals such as scattering. Thereafter, all spectra were normalized against the individual maximum. By preprocessing, we aim to investigate the comparability of different IR measurements under the best case; *i.e.*, with minimal influence from sample and non-absorptive contributions.

#### 2.3.2 Comparability across measurements

The comparability across measurements was investigated based on single-point IR spectroscopy, which was composed of one spectrum per measurement per substance. Ideally, all measurements are expected to output identical spectra for the same substance. In reality, multiple measurements always bear certain variations. Such variation can manifest as fluctuations in band intensity, band position, and bandwidth. It can also be identified from the decreased spectral similarity. All these facts laid the foundation for our analysis in this study.

As the first step, we calculated the spectral similarity across spectra from the same substance to gain a general idea of the spectra compatibility. Two commonly employed metrics for spectral similarity were used: the Euclidean distance (ED), for absolute similarity, and the Pearson's correlation coefficient (PCC)^37^ for relative similarity. In both cases, the calculation was conducted in three ways: between each spectral pair, between the overall mean spectrum and each single spectrum, and between the mean spectrum of the FTIR in transmission mode and each single spectrum. The third option was inspired by the fact that the FTIR in transmission mode remains the ‘gold standard’ IR technique with high stability and reliability. To make the interpretation easier, we used the difference 1-PCC instead of the original PCC so that larger values mean higher dissimilarity for both metrics.

For a deeper understanding, we investigated the changes in the intensity, width and position of selected IR bands. Only bands that are well distinguished and bear no shoulder bands were selected to make the calculation as precise as possible. We intentionally ensured the selection covered the full wavenumber range to gain a better view of the comparison. The reference positions of these bands were obtained from the average of O-PTIR-LS measurements using substrate CaF_2_. Details of the selected bands are provided in the next section. Specifically, the left and right boundaries of a band were calculated with the following steps. First, the spectrum was subjected to a smoothing filtering based on a Savitzky–Golay filter (window size of 5, order of 2).^38^ Based on the smoothed spectrum, the left and right boundary of the band was detected as the points where the intensity stops decreasing when going from the center to the side. The width of the band is defined as the larger half-width from the left or right side of the band, to avoid the influence of any shoulder band. The intensity and position were calculated from the maximum of the band, based on the spectrum without smoothing. After obtaining the intensities, widths, and positions for all selected IR bands, we transformed the absolute positions into relative shifts from the values of the O-PTIR-LS CaF_2_ measurement. This helps to address the different scales of the different bands so that they all fall into a ‘standardized’ range for easier visualization.

#### 2.3.3 Sample heterogeneity

As a cofactor of variability in IR spectroscopy, sample heterogeneity has to be well considered when comparing different measurements. To do so, we investigated the IR mappings from brain lipid and urea to calculate the within-mapping variability. The variation was benchmarked with both the 1-PCC and areas of selected IR bands. The 1-PCC was calculated between the mean spectrum of each mapping and the single spectrum within the same mapping. We did not calculate the band positions and widths because the spectra are observed to be of low quality and do not support precise band fitting. Imprecise band fitting can additionally introduce variations, which can bias the analysis. For the same reason, the band area instead of band intensity was used to ensure a higher precision of the calculation.

## 3. Results and discussion

With all steps and analysis introduced in the previous section, we present and discuss here the results obtained from the measurements in both single-point and mapping mode. The variability across different measurements (*i.e.*, cross-measurement variability) will be explored, unveiled, and interpreted in the former case. The analysis of mapping mode aims to rule out the effects of sample heterogeneity and further support the discoveries from the single-point measurements.

### 3.1 Results from single-point IR spectroscopy

The IR spectra after preprocessing are visualized in Fig. S1 for the single-point measurements of the different substances. The spectra look very similar in most cases, with obvious differences across measurements observed for the bands marked with arrows. In most cases, the LDIR measurement provided a clearly distinct IR spectrum compared to other measurements. Spectra appear more similar to each other when being measured with the same technique (*e.g.*, FTIR or O-PTIR). Particularly obvious variations are seen for both urea and glycogen within the spectral range of 1550–1800 cm^−1^, in addition to the region around 1100–1200 cm^−1^ of urea.

**Fig. 2:**
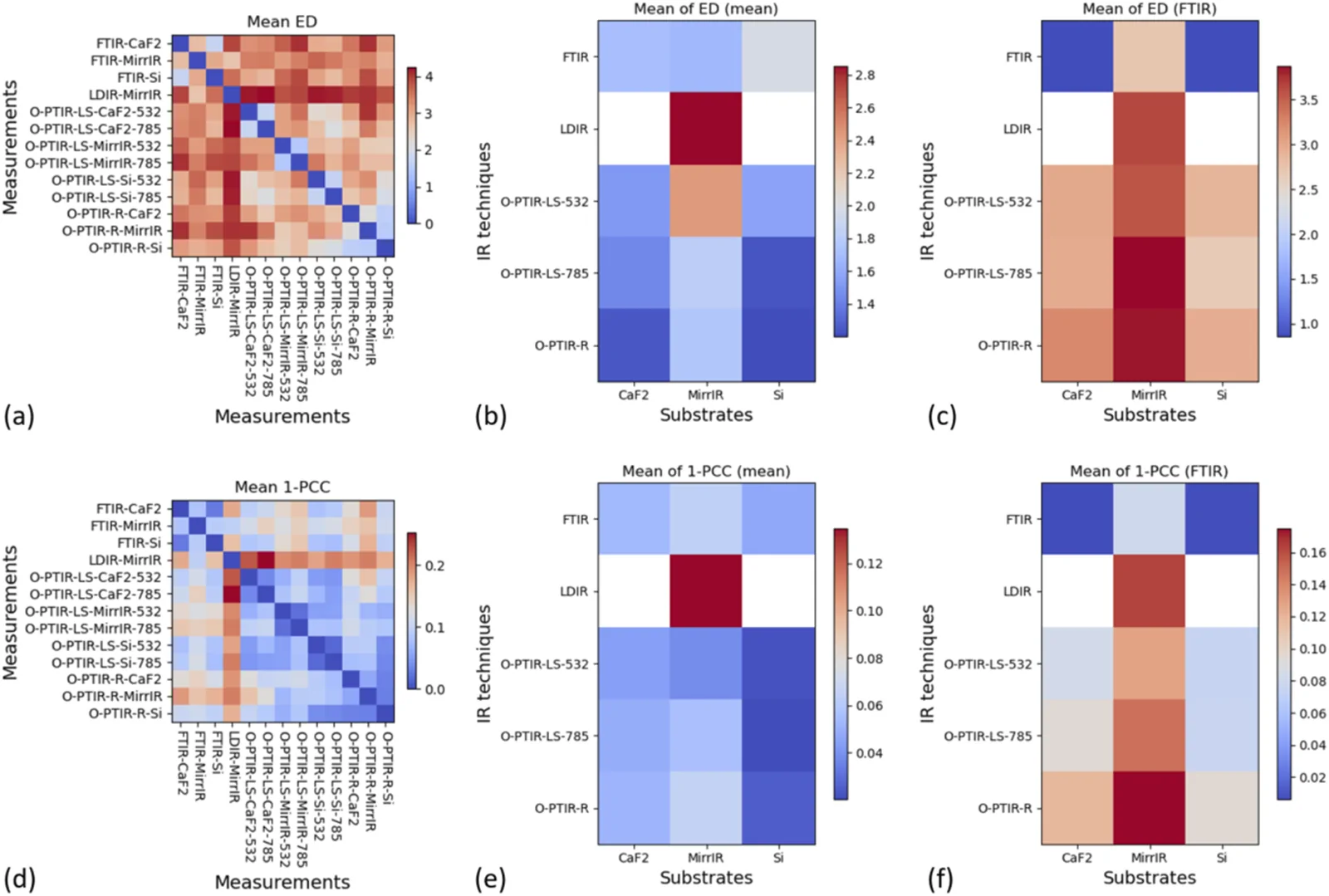
Results of spectral similarity based on the Euclidean distance (a–c) and the inverse-Pearson correlation coefficients (d–f). Both metrices were calculated in three manners: between spectral pairs (a and d), between the overall mean and a single spectrum (b and e), and between the mean of the FTIR transmission mode and a single spectrum (c and f).

To confirm these observations and go further, we approached the spectral similarity based on the ED and 1-PCC. The results for each substance are shown in Fig. S2–S4 and S5–S7 for the two metrics being calculated in the three manners, as previously mentioned. To make the description clearer, we condense these results into Fig. 2 by calculating the average of the same calculation across different substances. According to Fig. 2(a and d), which were calculated between each spectral pair, LDIR clearly differs from all other measurements. FTIR and O-PTIR differ slightly from each other, as demonstrated by the slightly larger values for both ED and 1-PCC between the two techniques than within them. Similarly, the calculations between the overall mean and a single spectrum, as given in Fig. 2(b and e), also demonstrated a clear separation between LDIR and the other two IR techniques. Further, the measurements on MirrIR substrate obviously differed from those on CaF_2_ and Si, as shown by both the highest ED and 1-PCC. Particularly, a larger ED was observed for the measurement from O-PTIR-LS-532 nm using MirrIR substrate. According to Fig. S3, this can be linked to the IR spectrum for brain lipid and lysozyme measured in this way, as is also marked in Fig. S1 with triangles. Measurements on CaF_2_ and Si bear slight differences in both ED and 1-PCC. The results look different when we used the mean spectrum of the FTIR transmission mode instead of the overall mean, as shown in Fig. 2(c and f). The reason is that the majority of the measurements were conducted with O-PTIR, so the overall mean is dominated by the O-PTIR. Nonetheless, the LDIR is still proven to be the most distinct technique compared with the other measurements. We could also say that MirrIR-based measurements are substantially different from those of the FTIR transmission mode, as supported by both larger ED and 1-PCC. A better comparability between FTIR and O-PTIR is maintained for CaF_2_ and Si. No clear difference was observed between CaF_2_ and Si in this case.

**Fig. 3:**
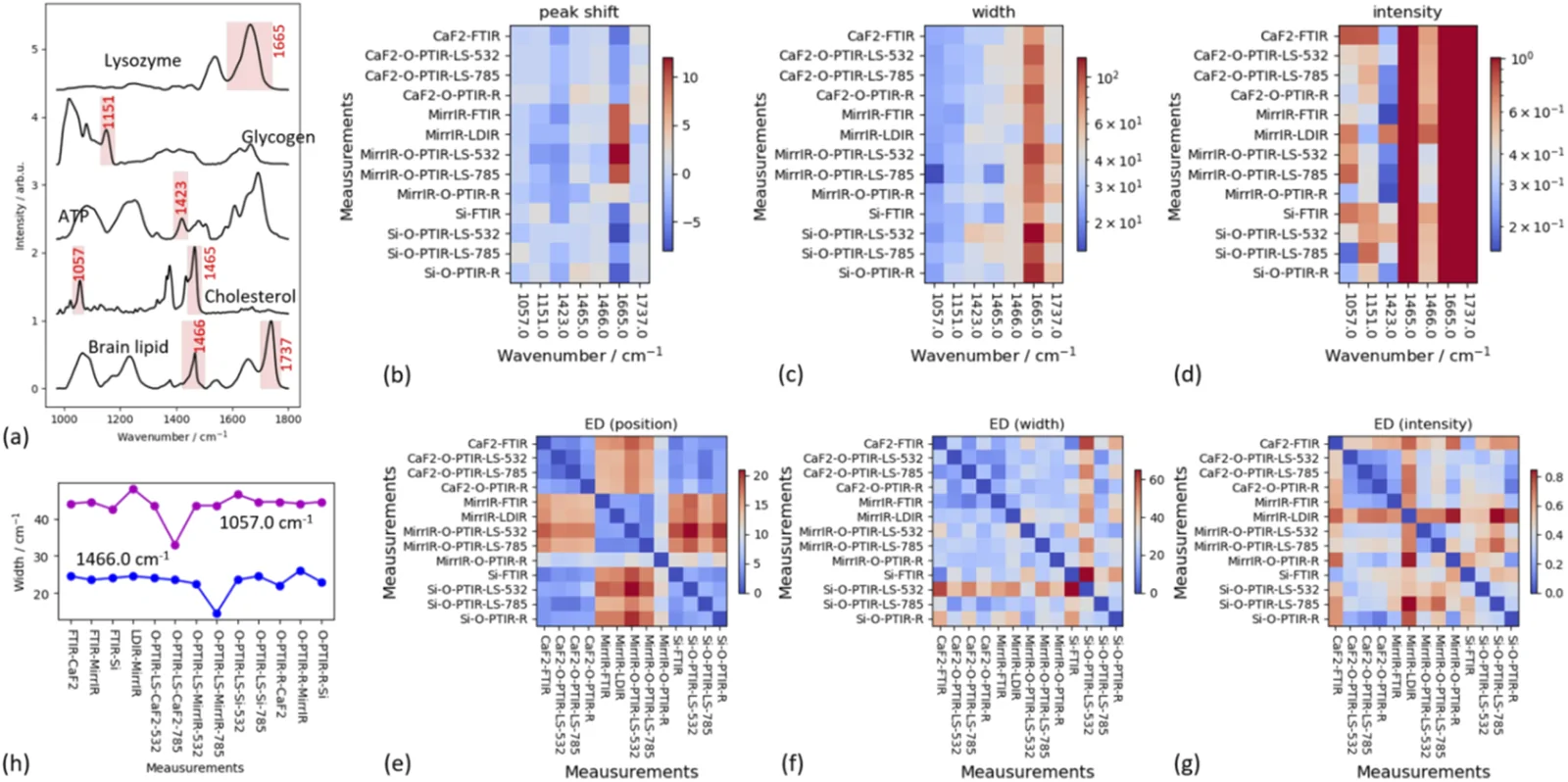
Results of band position, width, and intensity. In total, seven IR bands were selected from different substances, which are well distinguished from their neighboring bands, as shown in the subplot (a). The spectra from different substances are plotted with shifts on the intensity axis. Results of (b) band shift, (c) width, and (d) intensity plotted in detail for all IR bands and different measurements, with the *x*-axis denoting the reference position of each band and the *y*-axis showing the different measurements. Color bars of (c and d) are on a logarithmic scale. ED calculated for each measurement-pair based on the (e) resulting band positions, (f) widths, and (g) intensities. Notably, subplots (c and d) are on a logarithmic scale for better contrast. (h) Widths of two narrow bands from different measurements.

The variations in the different measurements were further investigated based on the band position, bandwidth, and band intensity. The calculations were performed on seven IR bands from different substances, of which the positions are marked on their respective mean spectrum in Fig. 3(a). The positions, widths, and intensities of all the bands under different measurements are shown in Fig. 3(b–d), where the *x*-axis denotes the reference position of each band and the *y*-axis demonstrates the measurements.

The position shift of each band is plotted in Fig. 3(b), with the deviations of the estimated position from the ‘reference’ positions shown on the *x*-axis. While most of the shifts are within ±1.5 cm^−1^, we can see the band at 1665 cm^−1^ is clearly blue-shifted for the MirrIR-based measurements and red-shifted for measurements with CaF_2_ and Si. To go a step further, we calculated the ED of the band shifts between each pair of measurements, leading to the results shown in Fig. 3(e). It is more obvious that the MirrIR-based measurements are distinguished from the others in respect of the band positions. Therein, we could see a correlation between the band position and the measurement substrate. To verify this observation, we performed a Mann–Whitney *U* test for each selected band and each substrate separately. The alternative hypothesis is that the width from a given substrate differs from the average width over all measurements. The resulting *p*-values are given in Fig. S8. Thereby, *p*-values are generally above 0.05, except for the two bands at 1151 and 1665 cm^−1^. This is probably the reason for the clustering behavior in Fig. 3(e). Despite not being significant for all bands, the results already suggested the critical role of substrate in the IR measurement. In fact, drop-casting substances on different substrates can produce substrate-dependent variations in morphology, crystallinity, thickness, and surface coverage that directly affect the measured spectra. As demonstrated in the bright-field images of samples dried on different substrates (Fig. S9), such morphological variations can significantly influence refractive index, optical scattering, effective optical path length, and consequently the observed infrared spectra. This could explain the appearance/disappearance of bands in different measurements (Fig. S1).

The widths and intensities are plotted in Fig. 3(c and d), with a color bar on a logarithmic scale for better contrast. On this basis, we calculated the ED between each pair of measurements for both metrics, leading to the results shown in Fig. 3(f and g). Accordingly, the widths and intensities of the bands appear to vary randomly across the measurements, without clear correlation to either measurement technique or substrate. To test the influence of spectral resolution on the peak width, we particularly investigated the width of two narrow bands at 1057 and 1066 cm^−1^, as the spectral resolution tends to affect narrower bands predominantly. As shown in Fig. 3(g), the widths of both bands demonstrate no clear difference between FTIR/LDIR and O-PTIR, where the spectral resolutions are different. This encouraged us to make comparisons with our current measurement settings.

### 3.2 Results from IR imaging

To verify the above observations, we investigated sample heterogeneity, particularly when compared to the cross-measurement variations. This was performed *via* the mapping of brain lipid and urea samples, of which the mean spectra and standard deviation are shown in Fig. S10 and S11 for all the different measurements. Subplots of each row correspond to different IR techniques, while each column gives spectra measured on a different substrate. At first glance, it is already clear that the measurements differ greatly with respect to the standard deviations; *i.e.*, the variability. CaF_2_ was shown to provide the most stable measurements among all three substrates, with the lowest standard deviations, followed by Si and MirrIR. Nevertheless, the small standard deviation of the FTIR-CaF_2_ measurement for both samples demonstrated low sample heterogeneity. Moreover, LDIR provides visually different spectra compared to all other measurements, and the spectra from MirrIR-based measurements bear visible differences from the other two substrates. These observations are further verified with detailed analysis based on band area and 1-PCC, as described in the next paragraph.

**Fig. 4:**
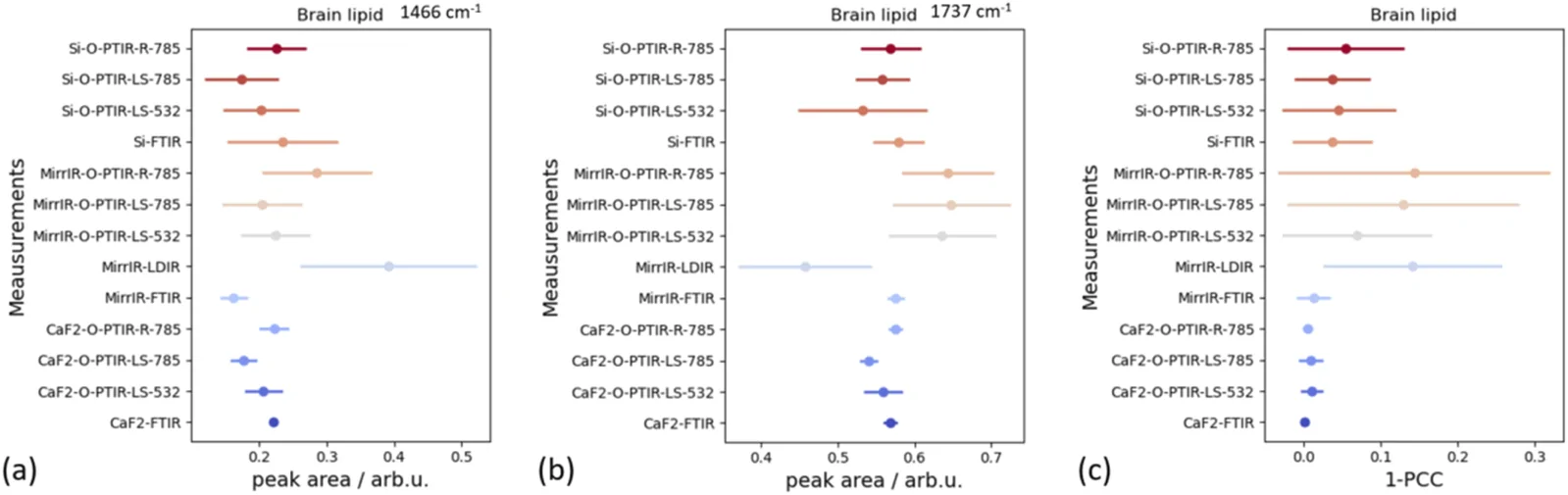
(a and b) Results of band area for the two bands at 1466 cm^−1^ and 1737 cm^−1^, respectively. (c) 1-PCC results.

As for the single-point measurement, the band area was calculated from IR bands that are well distinguished from their neighboring bands. Accordingly, we selected two bands from brain lipid and one from urea, as marked in Fig. S10 and S11. Here, the area instead of intensity was used to accommodate the noise in the mapping measurement. To do so, we integrated over the spectral range of 1420–1500 cm^−1^ and 1700–1760 cm^−1^ for the brain lipid and 1440–1490 cm^−1^ for the urea. The results are plotted as false-color images in Fig. S12–S14, in which the subplots are arranged by rows and columns for substrates and IR techniques, respectively. To facilitate understanding, we condensed the false-color plots as mean ± std (standard deviation) in Fig. 4(a–b) and S15(a) for the three bands. Like the spectral plots in Fig. S10 and S11, the different measurements bear varying standard deviation within the mapping, illustrating the variability in stability across the measurements. LDIR is consistently distinct from the other techniques. The variability appears to be more obvious across substrates than the IR techniques. This is seen not only from the standard deviation but also in the mean values of the band area: MirrIR-based measurements tend to have higher band areas and standard deviations than Si and CaF_2_. This could be attributed to the reflection effect caused by the MirrIR substrate, as was discussed previously. As a comparison, nonetheless, the sample heterogeneity remains an ignorable fact, as demonstrated by the small standard deviation within the CaF_2_-based mappings.

Similar phenomena occur for 1-PCC. Here again we used 1-PCC instead of PCC so that larger values represent larger variability. The detailed results for all mappings are shown as false-color images in Fig. S16 and S17, while the summarized mean ± std plots are shown in Fig. 4(c) and S13(b). The small standard deviation within the CaF_2_-based mappings again indicated sufficiently small sample heterogeneity. Again, the variability across substrates is more dominant than it is across IR techniques. The best stability of the CaF_2_-based measurements is proven by both the lowest mean values and the smallest standard deviations of 1-PCC, while the highest mean values and largest standard deviations indicate that the MirrIR-based measurements are the least stable.

Based on these IR mapping results, we conclude that sample heterogeneity is negligible compared to the variability across different IR techniques. Furthermore, the substrate influences band areas and spectral similarity more strongly than the IR technique. This is because these features are mainly controlled by how IR light interacts with the substrates. Differences in refractive index, optical path length, and reflection geometry determine the effective absorption strength. This means that transmission substrates, such as CaF_2_ and silicon, and reflective substrates, such as MirrIR, inherently produce different intensity patterns.^39–42^

### 3.3 Comparative interpretation of variability sources

In this study, we aim to illustrate and investigate the variability in the IR measurements with respect to IR techniques and substrates. The influence of sample heterogeneity was ruled out, based on the mapping results of brain lipid and urea samples. Surprisingly, the two sources contribute to the variability differently. Band position appeared to be more affected by substrates, as demonstrated in Fig. 3(e, g), 4(a–b), and S15(a). This can be the reason for the substrate-dependency pattern of the spectral similarity (see Fig. 4(c) and S15(b)). In contrast, the IR technique does not appear to affect spectral variability in a clear pattern. Except that LDIR differs substantially from both FTIR and O-PTIR. However, as LDIR was performed on MirrIR in transflection mode due to the technical limitations of the setup, we cannot clearly attribute the variability to LDIR itself, the substrate, or the measurement mode.

## 4. Conclusions

In this study, we investigated the comparability of IR measurements using three different IR techniques and three commonly used substrates. Six substances were analyzed in single-point mode, with two additionally measured in mapping mode. The spectral variability was benchmarked and illustrated from the metrics of band position, bandwidth, band intensity (area), and spectral similarity. In general, LDIR is demonstrated to be substantially different from FTIR and O-PTIR, while CaF_2_ provides generally the best stability within the investigated sample, followed by Si and MirrIR. With sample heterogeneity proven to be ignorable based on the analysis of the IR mapping, we separated the source of the variations into IR technique and substrate. Both sources were found to cause spectral variability in different ways. Despite the large variability across IR techniques, no clear pattern was observed between IR technique and the variability. The substrate, on the other hand, plays a critical role in band positions. This can be attributed to factors like sample-substrate refractive index match, which are critical for sample-substrate interface effects, including interference and reflection.

It should be noted that narrowing down the affecting factors to the two sources is a simplification of all the influential factors, as it is not possible to precisely disentangle all the different factors. For instance, to verify differences between reflection and transmission measurement modes would require measurements performed under comparable optical path lengths and interaction volumes using the same sample and substrate configuration. Such equivalence was not supported by the present experimental setups, and therefore this comparison is left for future work. With the results of this study, however, we could demonstrate that substantial spectral variability exists across different IR techniques and substrates, which should be considered in real applications. Approaches, whether hardware- or algorithm-based, are required to standardize the measurements, making them more comparable.

## Conflicts of interest

There are no conflicts of interest to declare.

## Supplementary Material

RA-OLF-D6RA03641D-s001

## Data Availability

Data are available in the manuscript, supplementary information (SI), or from the corresponding author upon reasonable request. Supplementary information is available. See DOI: https://doi.org/10.1039/d6ra03641d.
