# Supplementary material for: Comparability across infrared spectroscopy techniques and substrates for bioanalytical applications
Source: RSC Adv. 2026 Aug 27;16(42):50783–92. doi: 10.1039/d6ra03641d (PMC13519905; doi:10.1039/d6ra03641d)
Supplement: Supplementary file 1 [file RA-016-D6RA03641D-s001.pdf]

Supplementary Information (SI) for RSC Advances.
This journal is © The Royal Society of Chemistry 2026

Comparability across Infrared Spectroscopy Techniques and Substrates for bioanalytical applications

Shuxia Guo[1, 2 *], Supatcharee Cael[2], Samir F. El-Mashtoly[1, 2], Christoph Krafft[2], Jürgen Popp[1, 2], Thomas Bocklitz[1, 2]

[1] Institute of Physical Chemistry (IPC) and Abbe Center of Photonics (ACP), Friedrich Schiller University Jena, Member of the Leibniz Centre for Photonics in Infection Research (LPI), Helmholtzweg 4, 07743 Jena, Germany

[2] Leibniz Institute of Photonic Technology, Member of Leibniz Health Technologies, Member of the Leibniz Centre for Photonics in Infection Research (LPI), Albert-Einstein-Straße 9, 07745 Jena, Germany

*Correspondence: shuxia.guo@leibniz-ipht.de

Table S1. Summary of abbreviations in the manuscript, ordered according to their appearance in the manuscript.

Abbreviations	Full Name
IR	Infrared (spectroscopy)
FTIR	Fourier transform infrared spectroscopy
O-PTIR	Optical photothermal infrared spectroscopy
LDIR	Laser direct infrared spectroscopy
QCLs	Quantum cascade lasers
ATR	Attenuated total reflectance
RI	Refractive index
Si	Silicon
ED	Euclidean distance
PCC	Pearson's correlation coefficients

Table S2. IR bands of involved substances based on O-PTIR LS MirrIR-785 on substrate CaF_2

Substance	Bands (cm^{-1})
ATP	1047, 1104, 1175, 1217, 1266, 1330, 1420, 1480, 1505, 1577, 1606, 1656, 1691
Brain lipid	1064, 1144, 1172, 1228, 1378, 1414, 1466, 1491, 1539, 1655, 1735
Cholesterol	985, 1007, 1023, 1056, 1083, 1108, 1130, 1141, 1158, 1190, 1220, 1238, 1254, 1271, 1304, 1333, 1366, 1375, 1433, 1465, 1536, 1632, 1670, 1730
Glycogen	1030, 1080, 1151, 1201, 1330, 1365, 1412, 1459, 1600, 1627, 1665
Lysozyme	1172, 1228, 1386, 1442, 1455, 1537, 1620, 1661

Urea	1465, 1586, 1613, 1623, 1630, 1649
ED	Euclidean distance
PCC	Pearson’s correlation coefficients

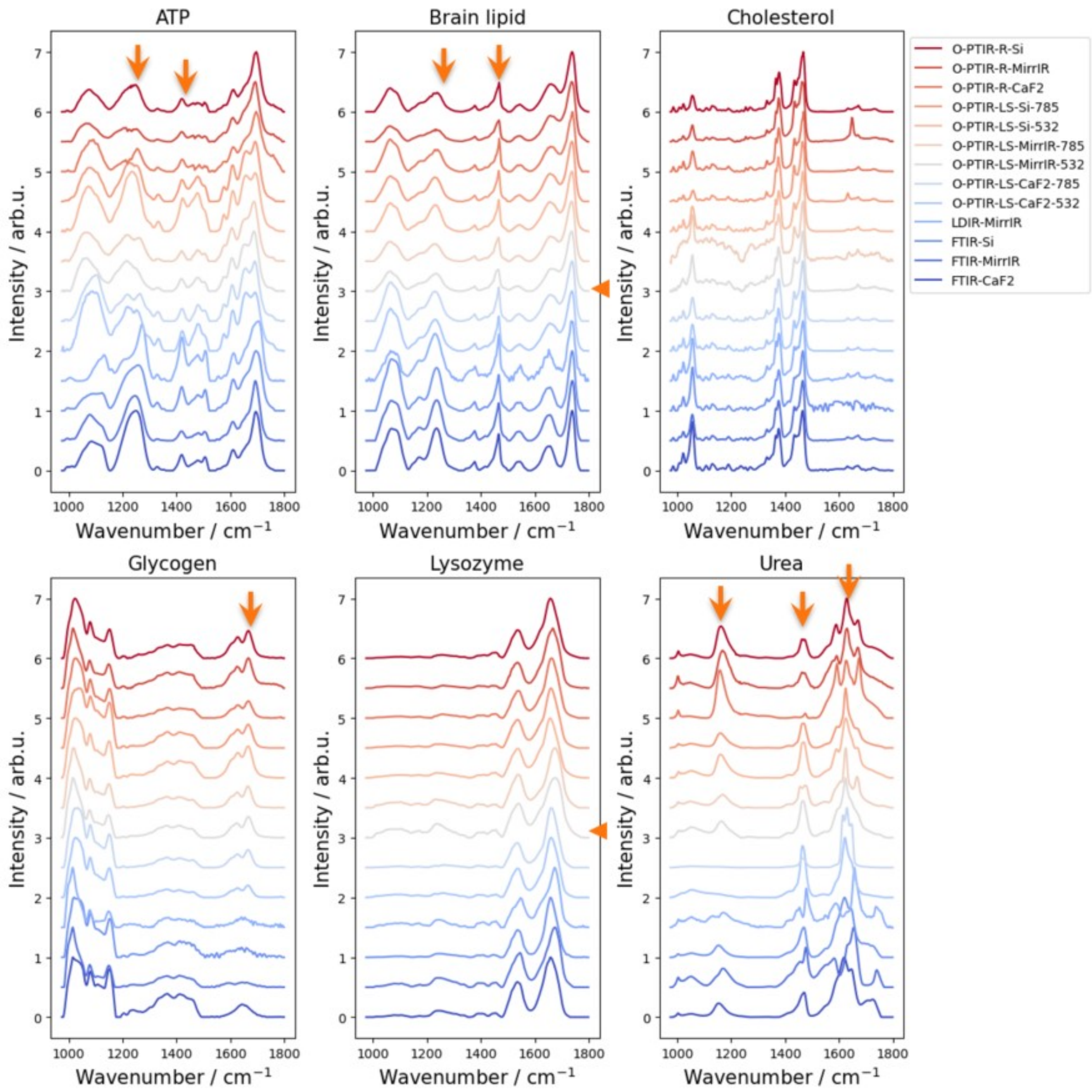

Figure S1. Spectra different measurements from different substances.

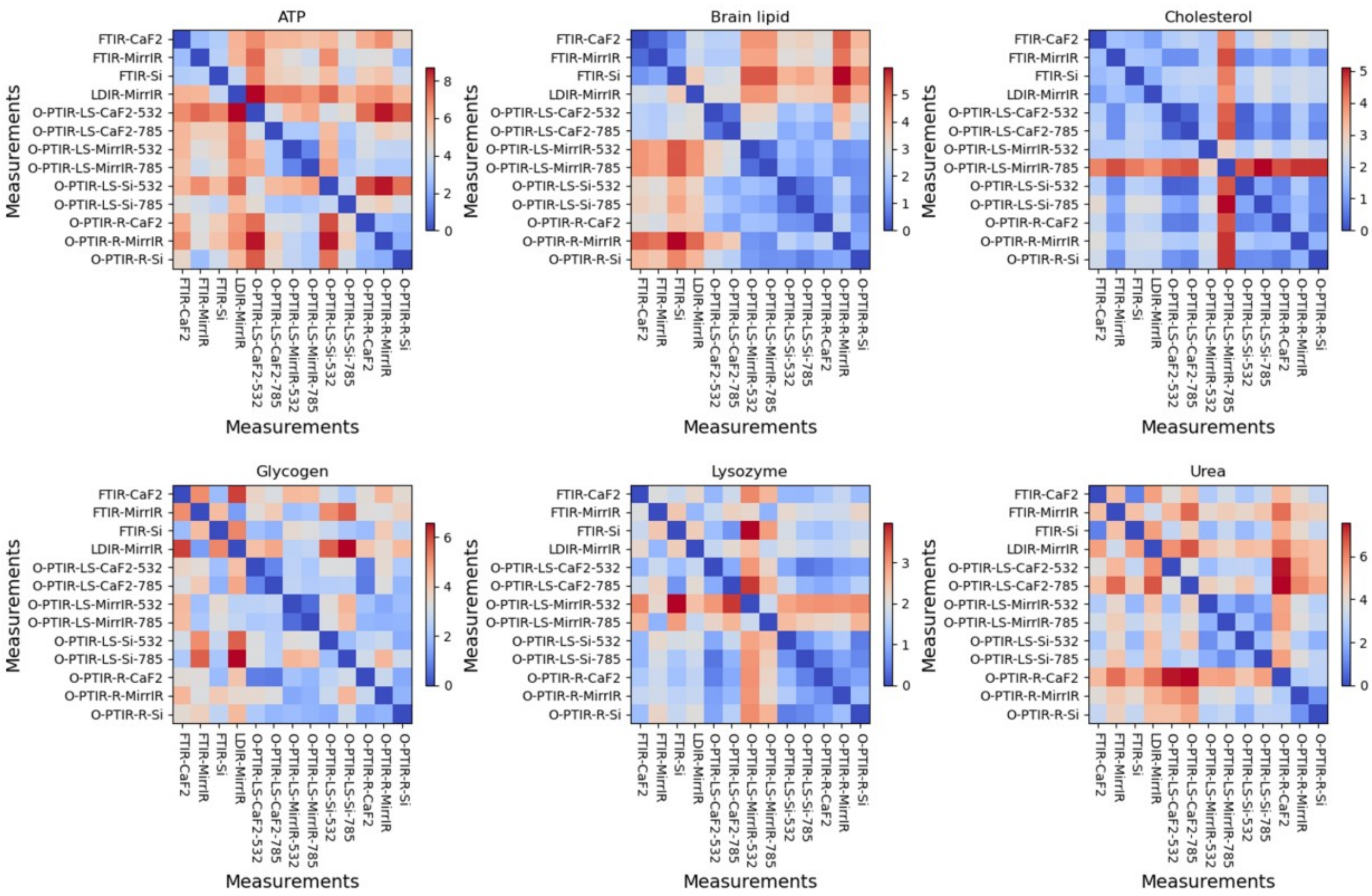

Figure S2. Results of Euclidean distance (ED) calculated between each spectral pair of the different measurements for the same substance.

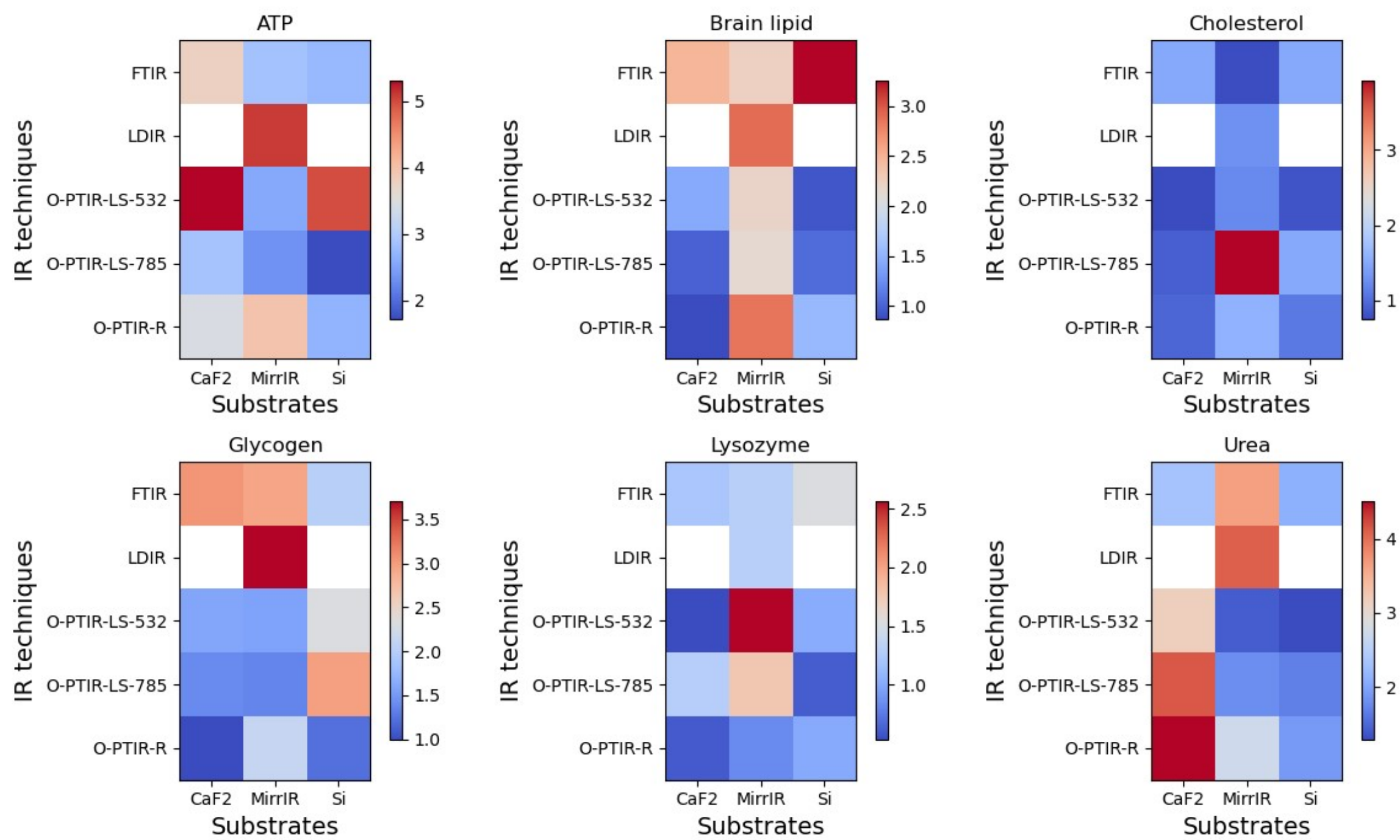

Figure S3. Results of Euclidean distance (ED) calculated between the overall mean and each measurement for the same substance.

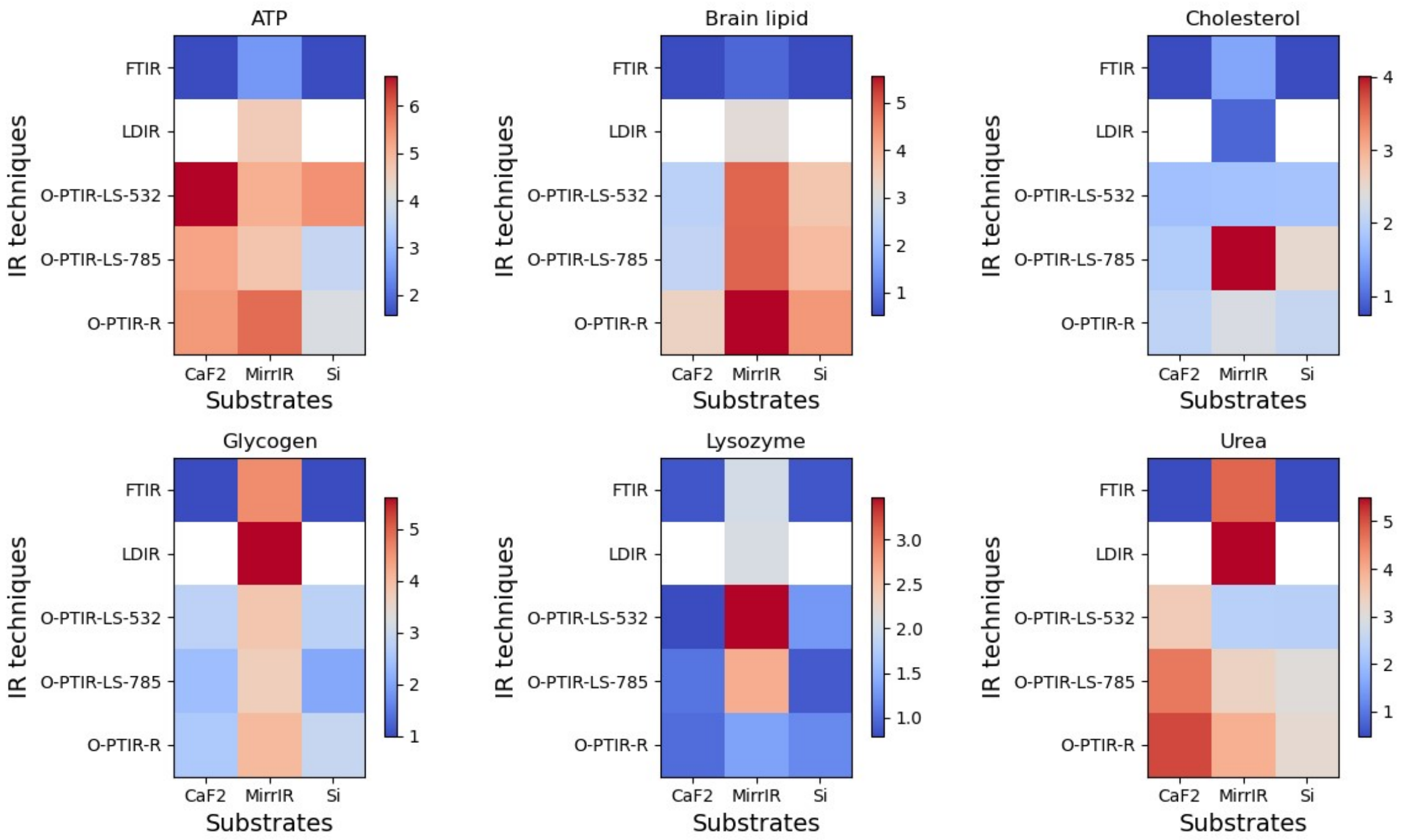

Figure S4. Results of Euclidean distance (ED) calculated between the mean of FTIR transmission mode and each measurement for the same substance.

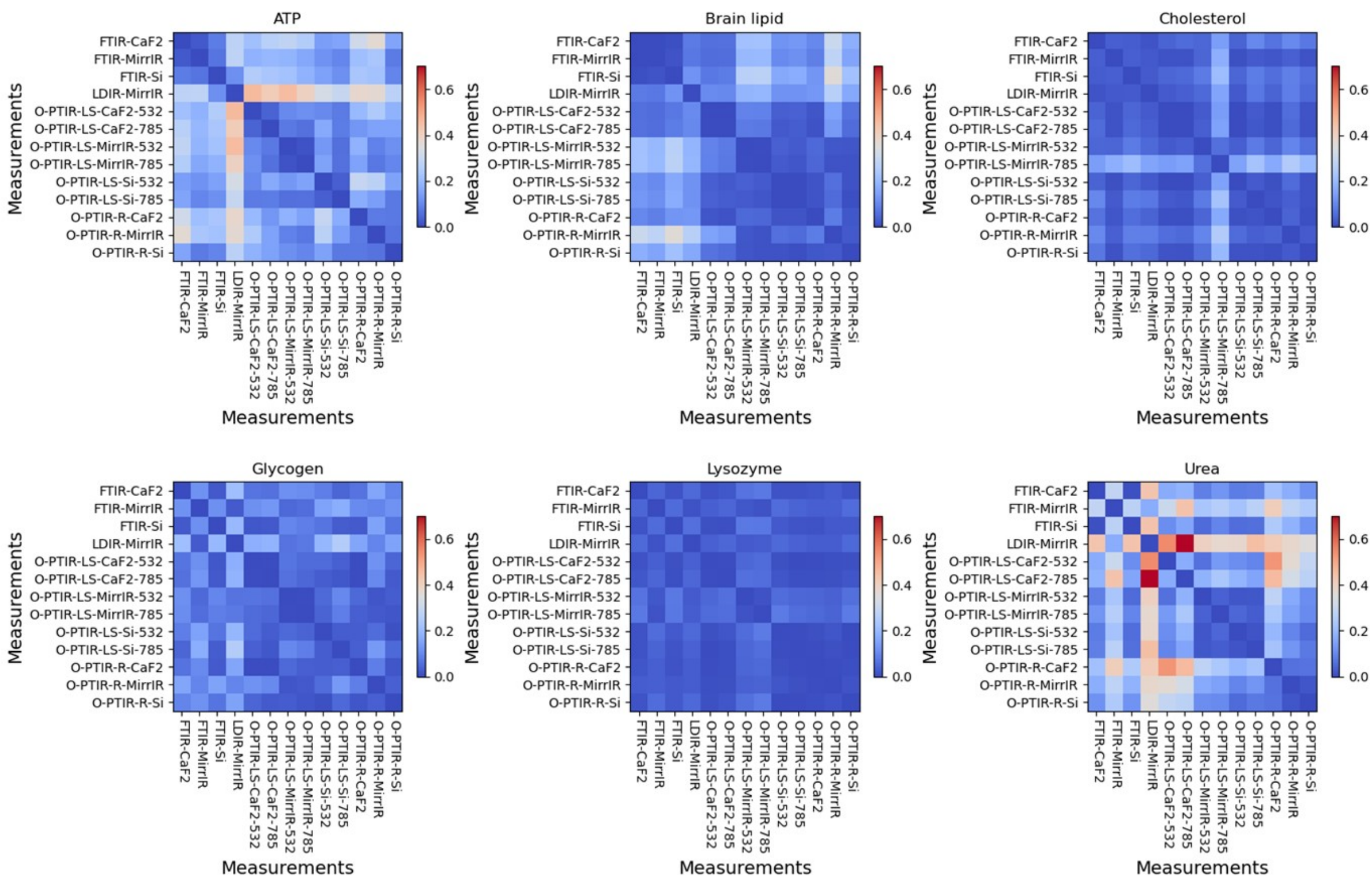

Figure S5. Results of inversed Pearson's correlation coefficients (PCC) calculated between each spectral pair of the different measurements for the same substance.

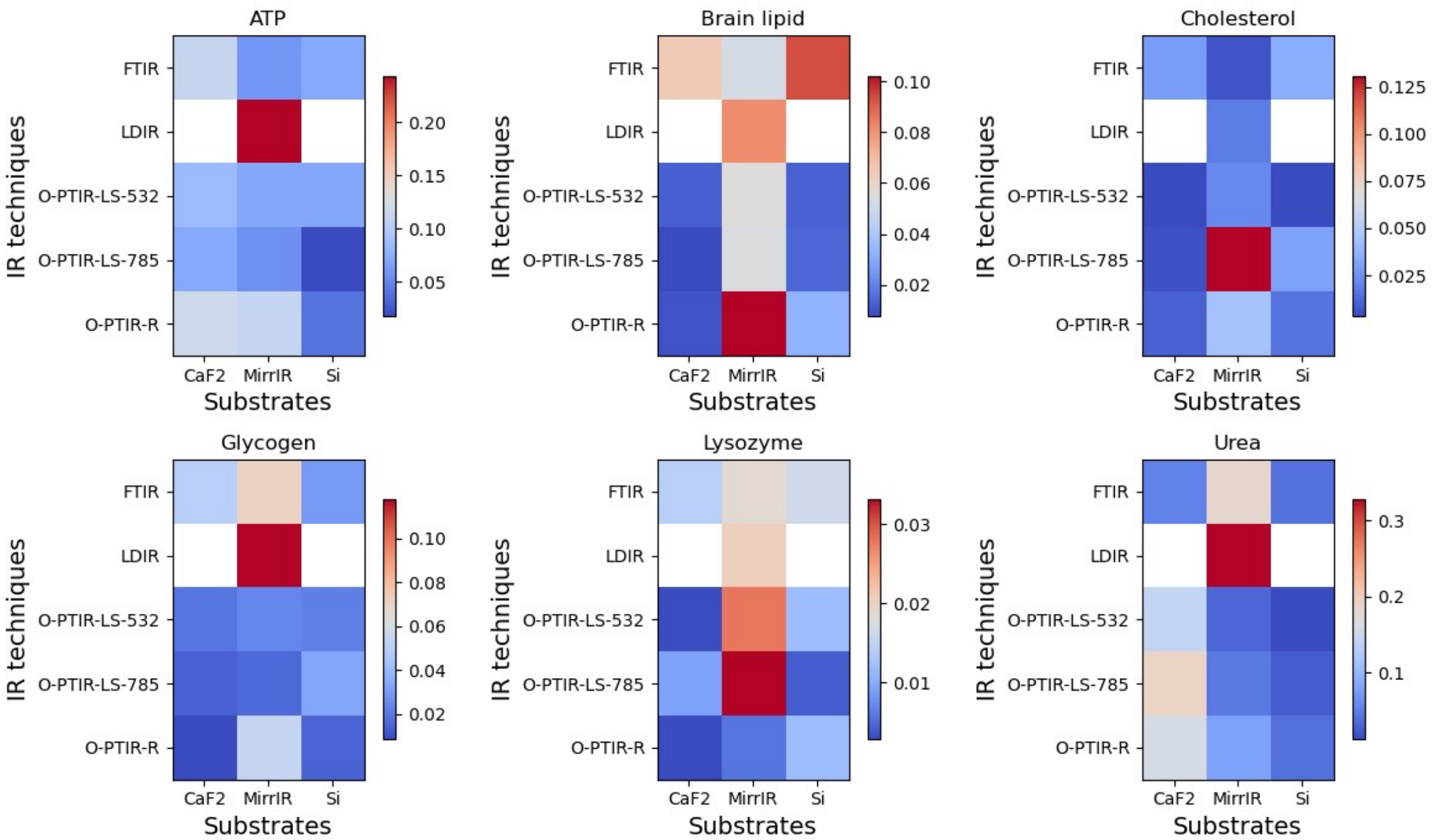

Figure S6. Results of inversed Pearson's correlation coefficients (PCC) calculated between the overall mean and each measurement for the same substance.

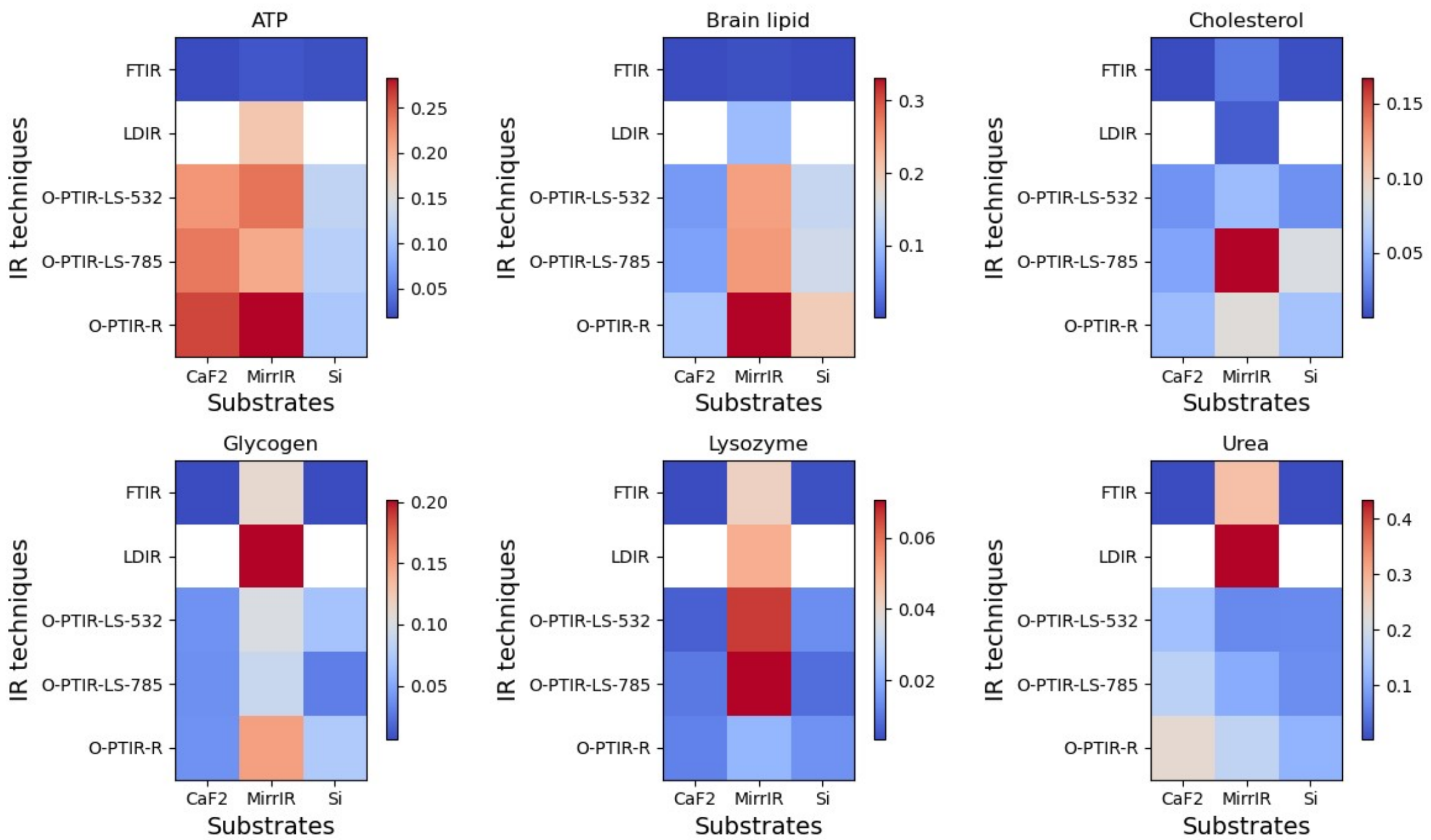

Figure S7. Results of inversed Pearson's correlation coefficients (PCC) calculated between the mean of FTIR transmission mode and each measurement for the same substance.

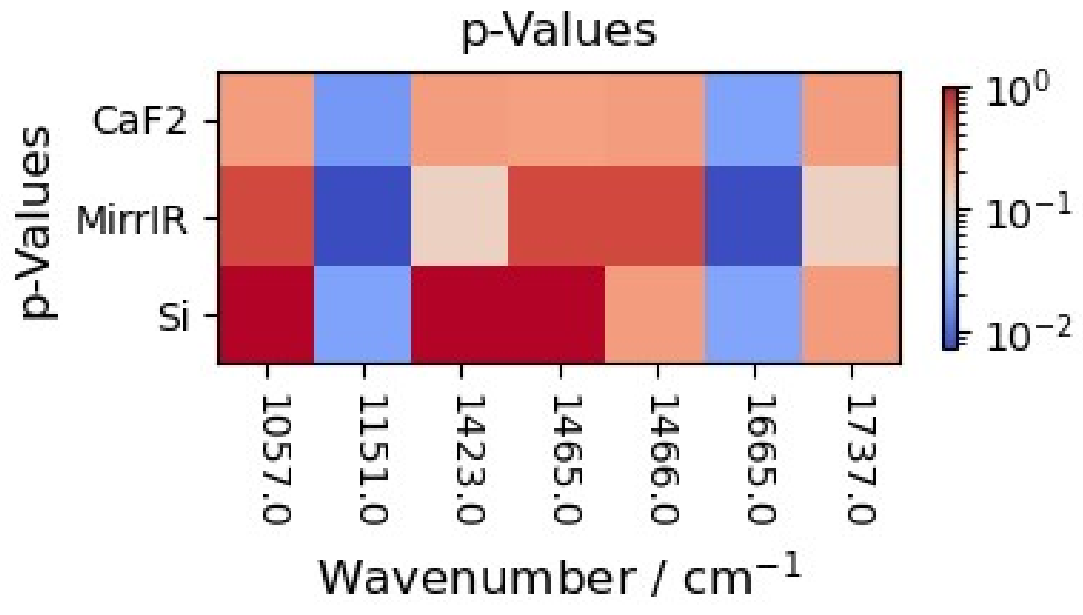

Figure S8 p-Valuses of Mann-Whitney U test. The test was done separately for each band and each substrate separately. Thereby the alternative hypotheis is that the width of each substrate differe from the average width over all measurements.

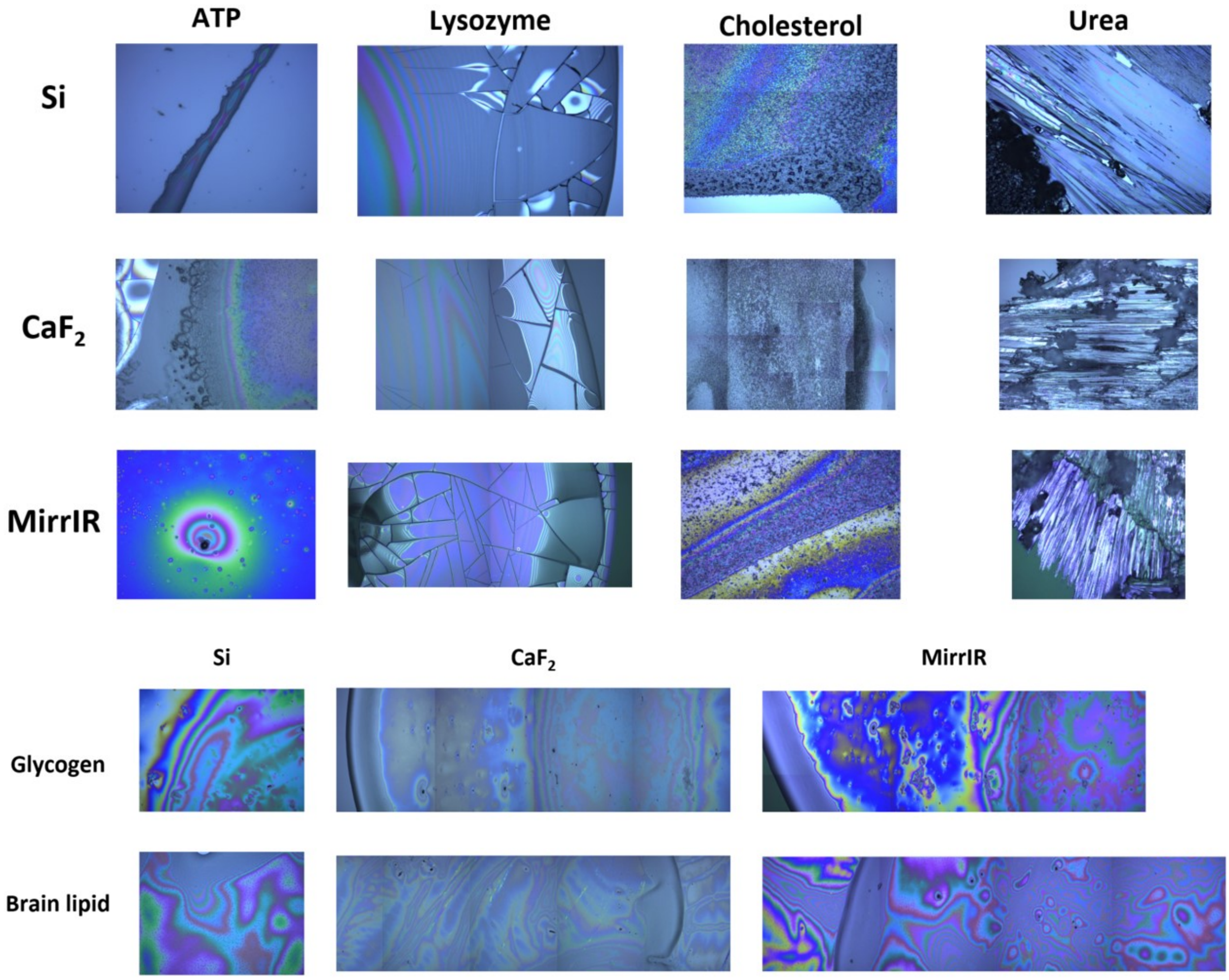

Figure S9. Bright field image of substances on the three substrates. Image acquired with OPTIR-R setup.

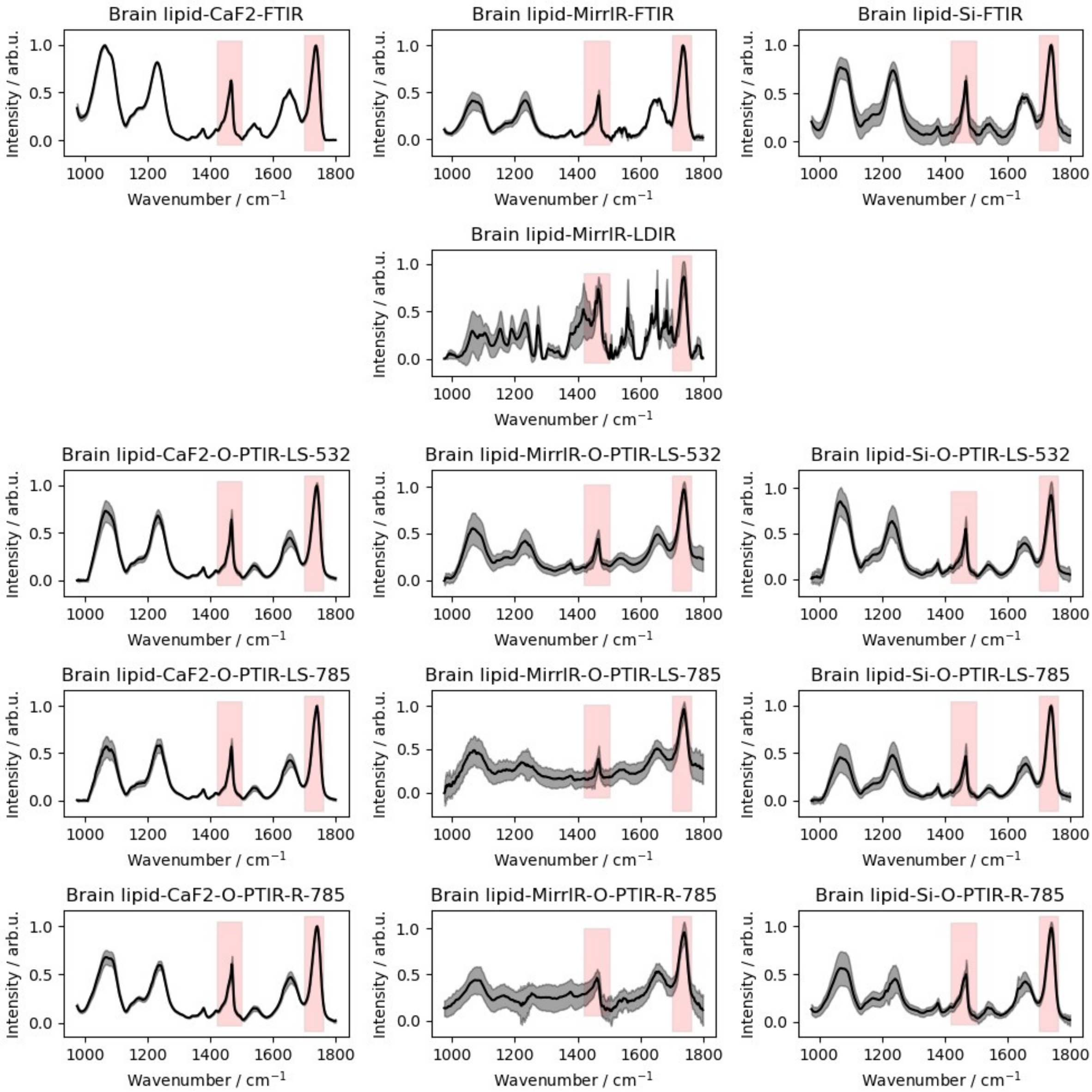

Figure S10. Mean and standard deviation of the IR mappings of different measurements from brain lipids. Highlighted bands are those selected for the calculation of peak area.

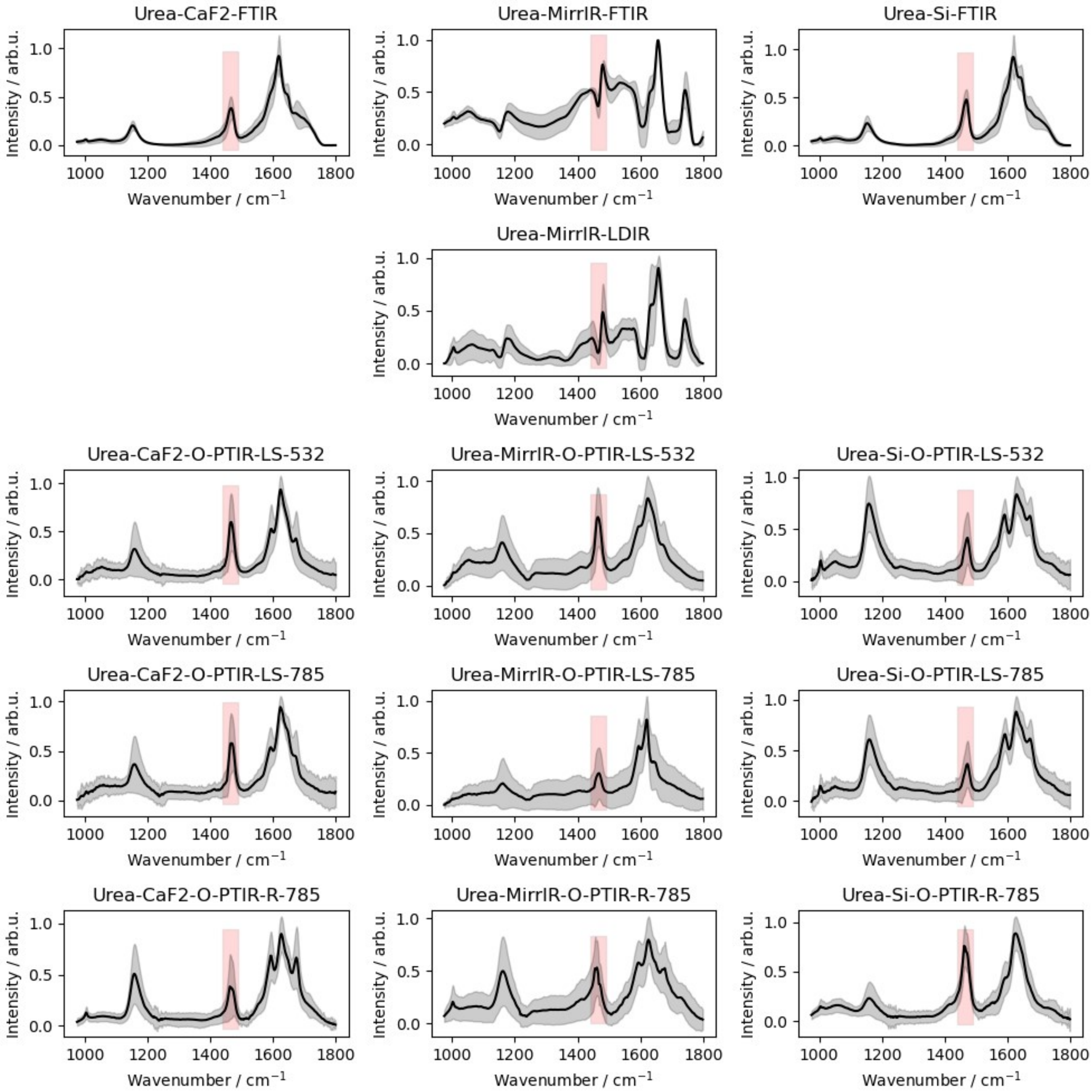

Figure S11. Mean and standard deviation of the IR mappings of different measurements from Urea. Highlighted bands are those selected for the calculation of peak area.

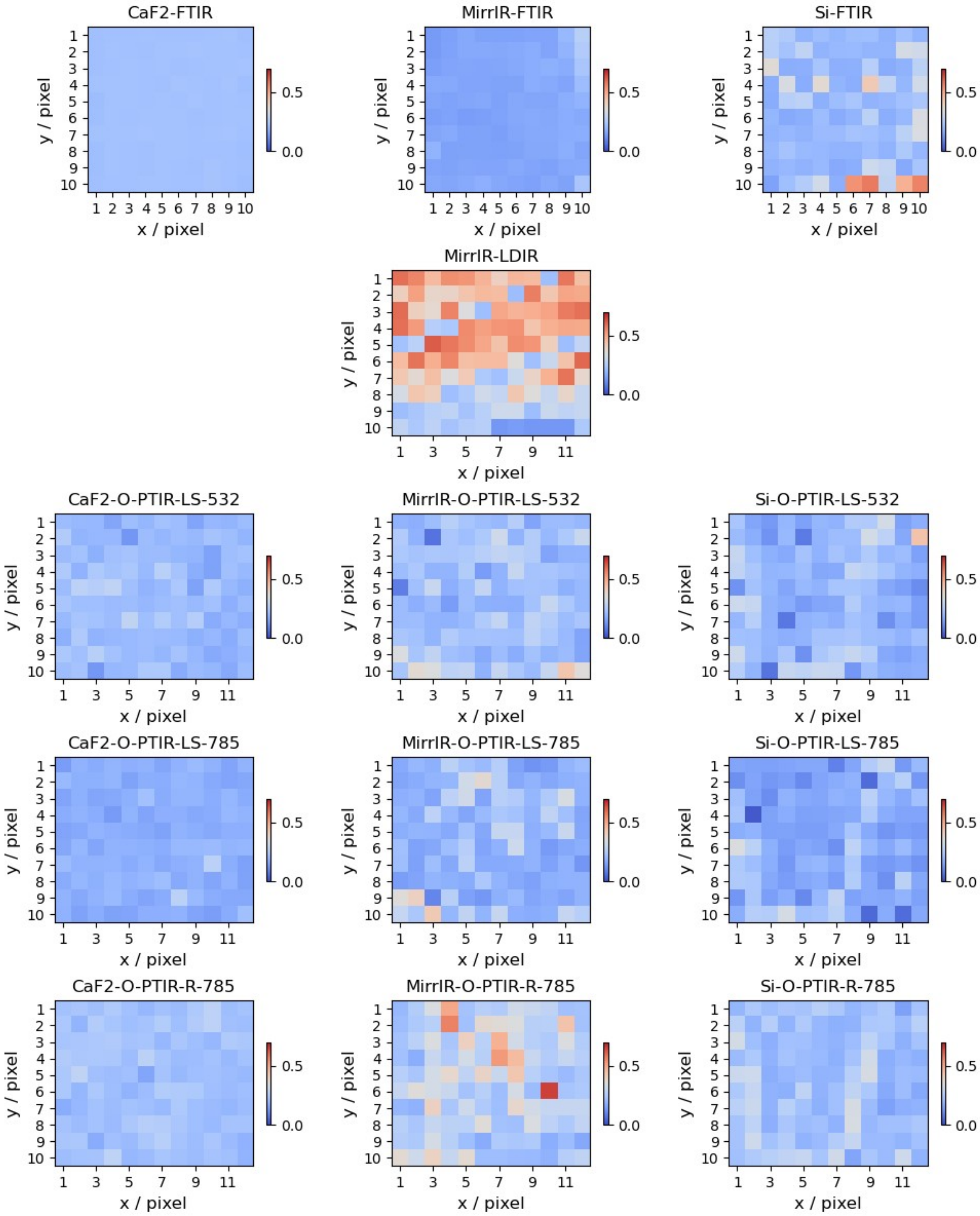

Figure S12. Area of the band at 1466 cm^{-1} for the IR mappings of different measurements from brain lipids. X- and y-axis provide the pixel index of the measurement.

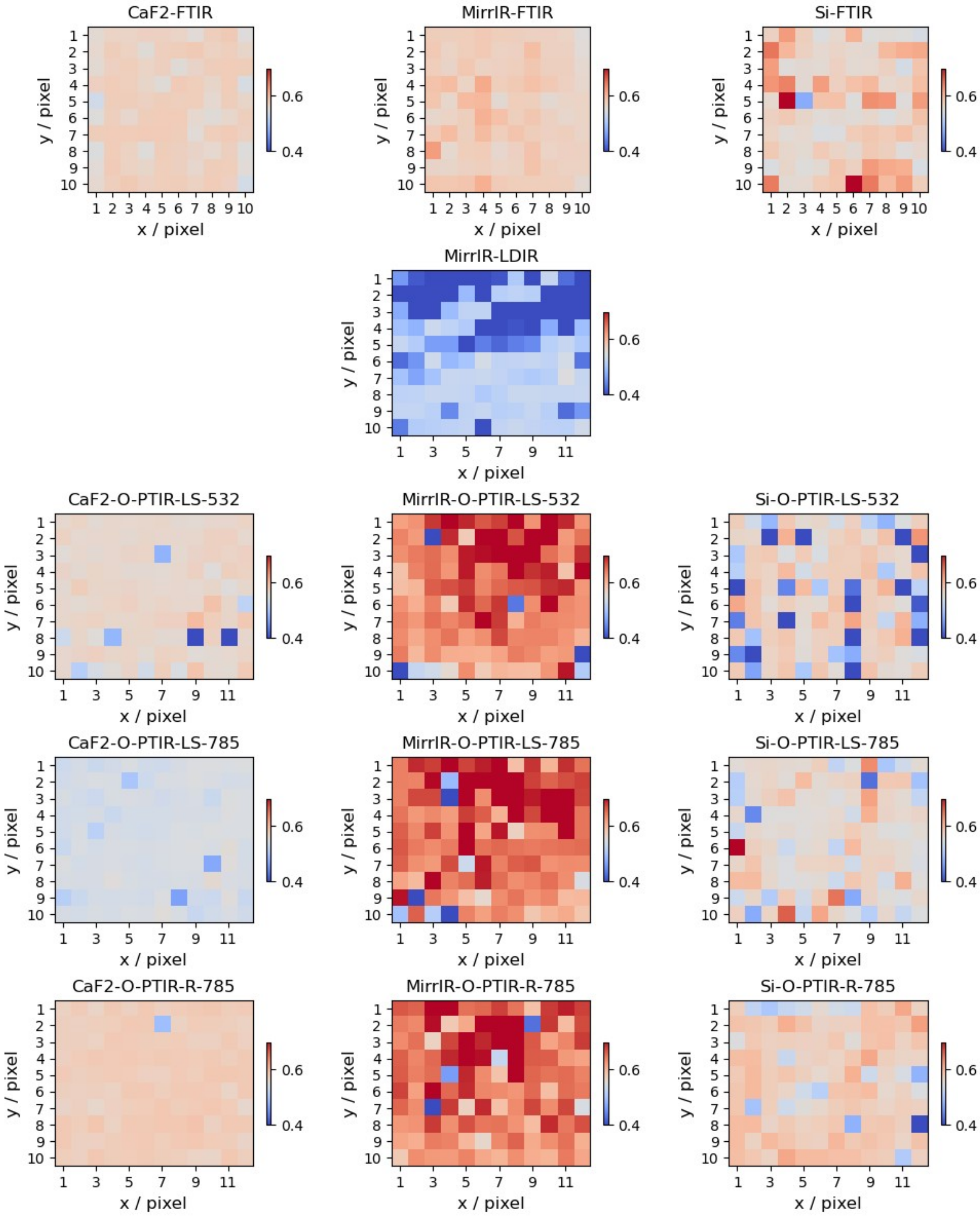

Figure S13. Area of the band at 1737 cm^{-1} for the IR mappings of different measurements from brain lipids. X- and y-axis provide the pixel index of the measurement.

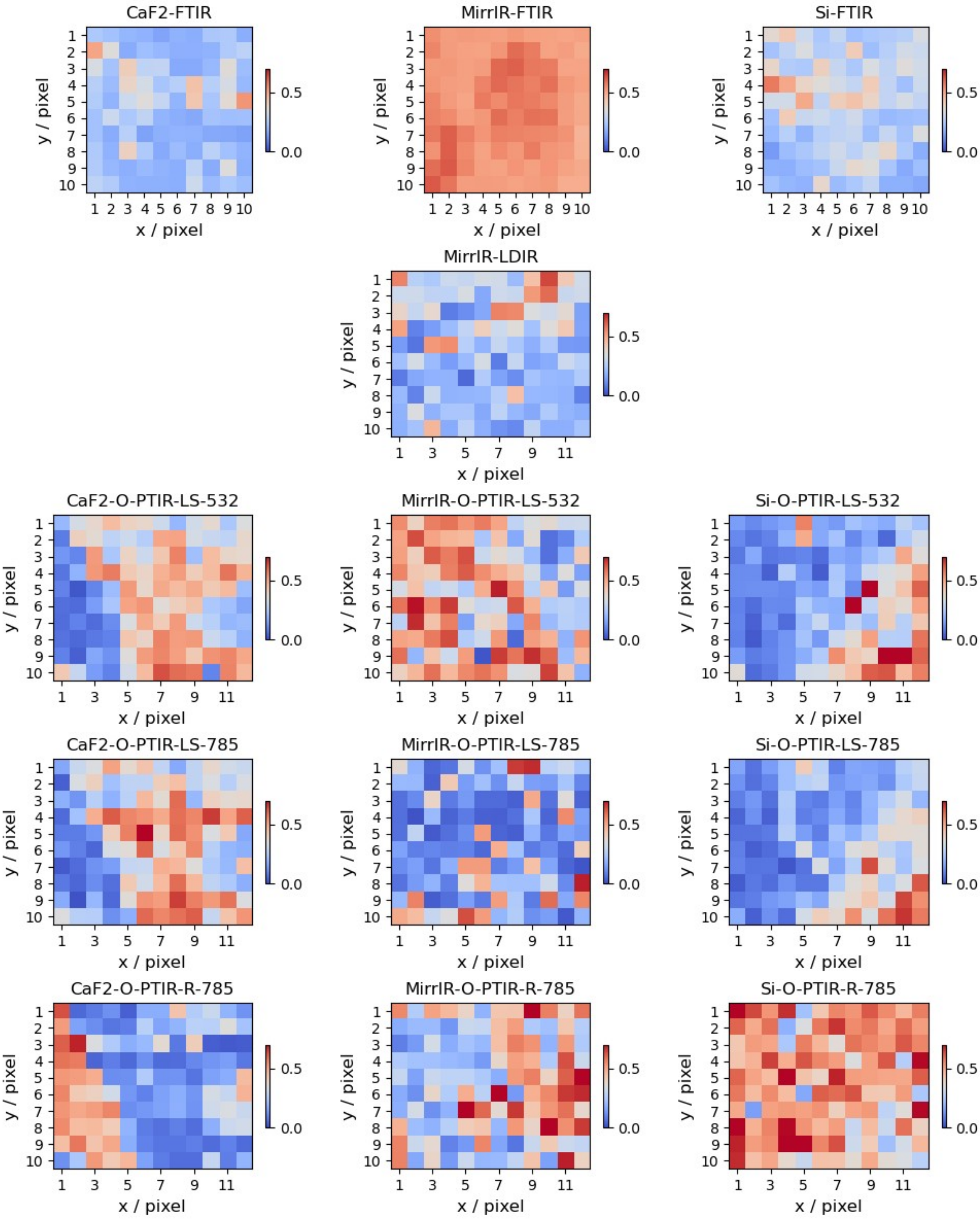

Figure S14 Area of the band at 1467 cm^{-1} for the IR mappings of different measurements from urea. X- and y-axis provide the pixel index of the measurement.

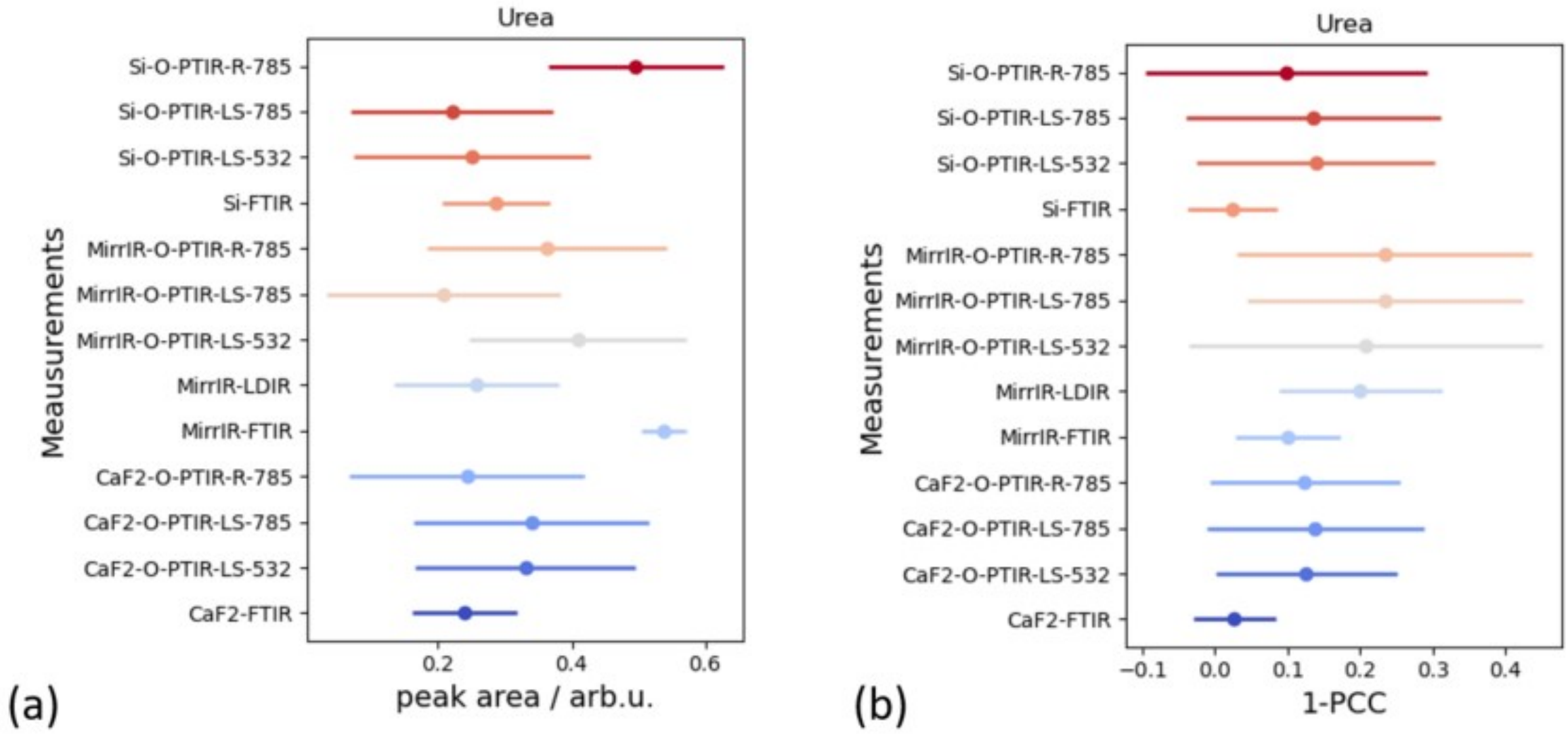

Figure S15. Results of peak area and inversed PCC from the mapping of urea. Different measurements are visualized individually as $mean \pm std$, i.e., mean and standard deviation over values of all spectra included in the corresponding mapping.

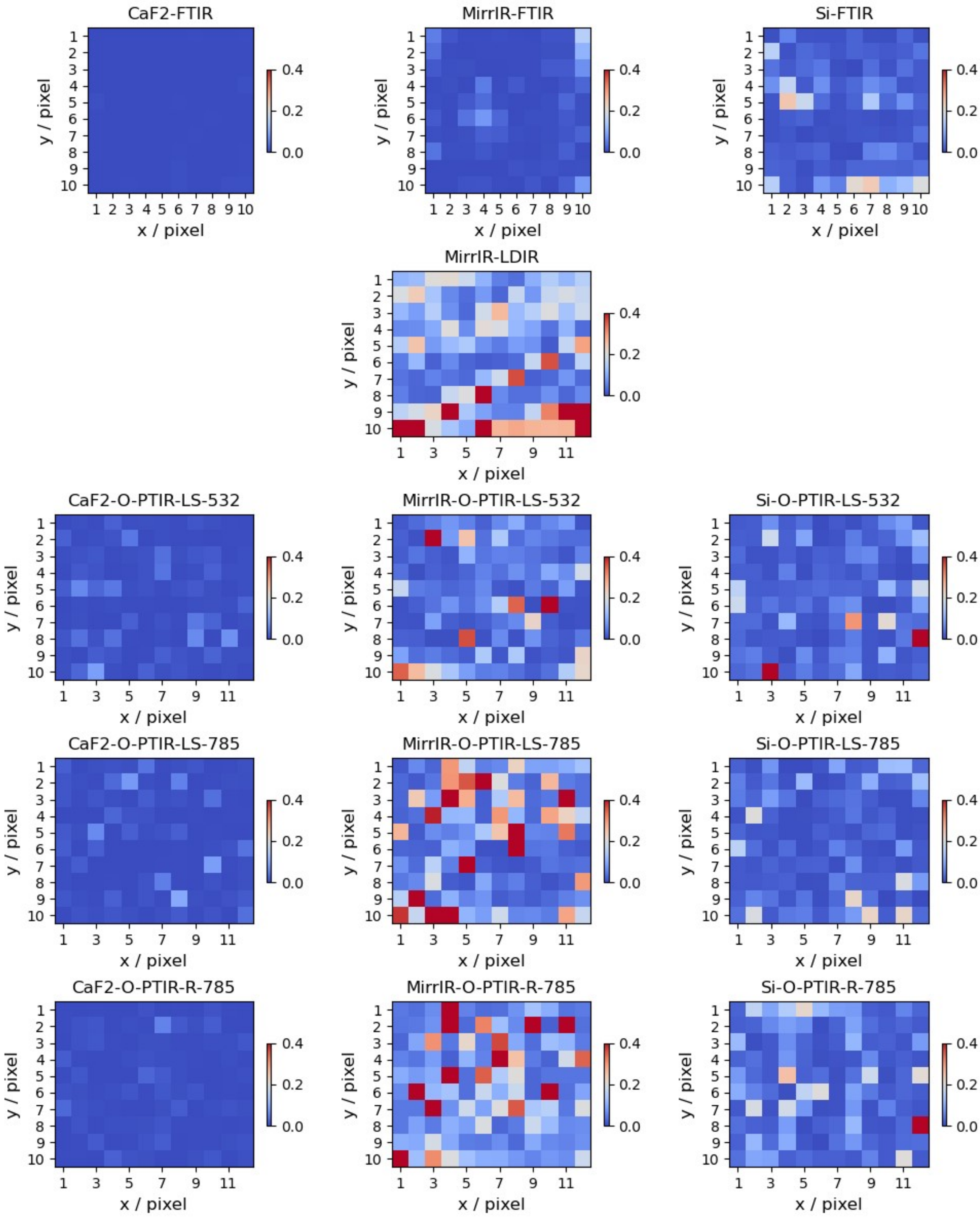

Figure S16. I-PCC from the IR mappings of different measurements from brain lipids.

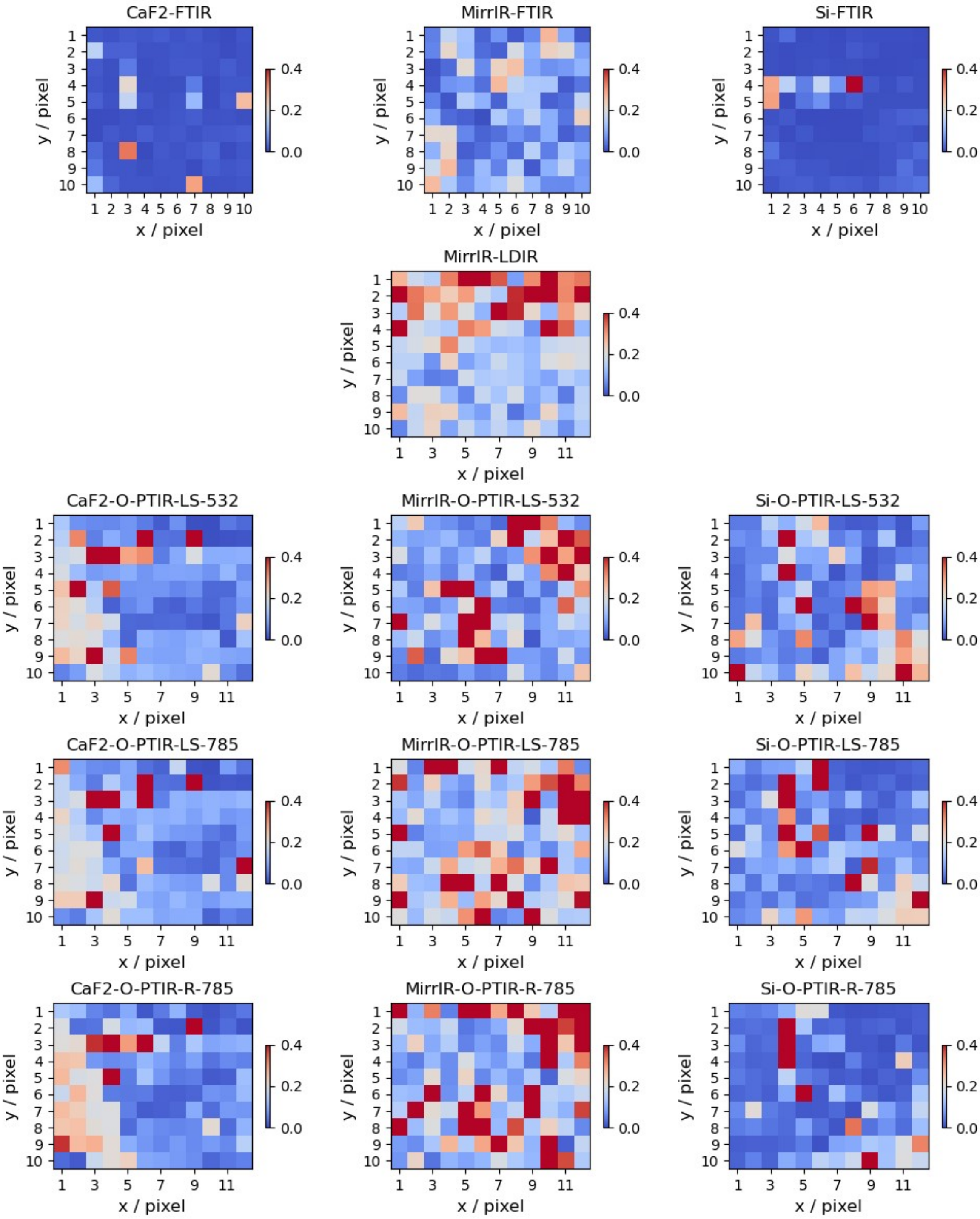

Figure S17. I-PCC from the IR mappings of different measurements from urea
